# Thermal Transport Characteristics of Human Skin Measured *In Vivo* Using Ultrathin Conformal Arrays of Thermal Sensors and Actuators

**DOI:** 10.1371/journal.pone.0118131

**Published:** 2015-02-06

**Authors:** R. Chad Webb, Rafal M. Pielak, Philippe Bastien, Joshua Ayers, Juha Niittynen, Jonas Kurniawan, Megan Manco, Athena Lin, Nam Heon Cho, Viktor Malyrchuk, Guive Balooch, John A. Rogers

**Affiliations:** 1 Frederick Seitz Materials Research Laboratory, Department of Materials Science and Engineering, University of Illinois at Urbana-Champaign, Urbana, Illinois, United States of America; 2 L’Oréal California Research Center, San Francisco, California, United States of America; 3 L’Oréal Research and Innovation, Aulnay sous Bois, France; 4 Tampere University of Technology, Department of Electronics and Communication Engineering, Korkeakoulunkatu 3, Tampere, Finland; 5 L’Oréal Early Clinical, Clark, New Jersey, United States of America; 6 L’Oréal Digital Incubator, Clark, New Jersey, United States of America; Texas A&M University, UNITED STATES

## Abstract

Measurements of the thermal transport properties of the skin can reveal changes in physical and chemical states of relevance to dermatological health, skin structure and activity, thermoregulation and other aspects of human physiology. Existing methods for *in vivo* evaluations demand complex systems for laser heating and infrared thermography, or they require rigid, invasive probes; neither can apply to arbitrary regions of the body, offers modes for rapid spatial mapping, or enables continuous monitoring outside of laboratory settings. Here we describe human clinical studies using mechanically soft arrays of thermal actuators and sensors that laminate onto the skin to provide rapid, quantitative *in vivo* determination of both the thermal conductivity and thermal diffusivity, in a completely non-invasive manner. Comprehensive analysis of measurements on six different body locations of each of twenty-five human subjects reveal systematic variations and directional anisotropies in the characteristics, with correlations to the thicknesses of the epidermis (EP) and stratum corneum (SC) determined by optical coherence tomography, and to the water content assessed by electrical impedance based measurements. Multivariate statistical analysis establishes four distinct locations across the body that exhibit different physical properties: heel, cheek, palm, and wrist/volar forearm/dorsal forearm. The data also demonstrate that thermal transport correlates negatively with SC and EP thickness and positively with water content, with a strength of correlation that varies from region to region, e.g., stronger in the palmar than in the follicular regions.

## Introduction

Skin is the largest organ of human body and it provides one of the most diverse sets of functions. The outermost layer, the stratum corneum (SC), serves as a protective barrier and the first defense against physical, chemical and biological damage. The skin also receives and processes multiple sensory stimuli, such as touch, pain and temperature and aids in the control of body temperature and the flow of fluids in/out of the body [1]. These processes are highly regulated by nervous and circulatory systems, but also depend directly and indirectly on thermal characteristics. The thermal transport properties of this tissue system can reflect physical/chemical states of the skin, with potentially predictive value in contexts ranging from dermatology to cosmetology. Measurement systems for *ex vivo* analysis [2,3] have some utility in establishing a general understanding of the properties, but they are irrelevant to investigations of the skin as an integral part of a complex, living organism. Existing *in vivo* approaches couple the use of laser heating or induced changes in the temperature of the ambient with infrared thermography [4–6], or they exploit rigid probes that press against the skin [7,8]. These and other previously reported methods only apply to certain regions of the skin; they do not readily allow thermal mapping measurement or determination of anisotropic properties and they operate effectively only in controlled, laboratory settings. As a result, little information exists that quantitatively characterizes the relationships between the *in vivo* thermal transport properties of skin and clinically relevant parameters such as hydration, vascularization and structure. Here, we introduce strategies that exploit ultrathin, soft systems [9–18] of thermal actuators and sensors for robust, precise transport measurements, in a non-invasive manner that can rapidly capture both orientation and position dependent characteristics. Assessments of the skin at six different body locations in twenty-five human subjects illuminate systematic variations in both the thermal conductivity and thermal diffusivity, for which measurements by optical coherence tomography (OCT), and electrical impedance yield additional insights into the underlying physiology.

**Fig 1:**
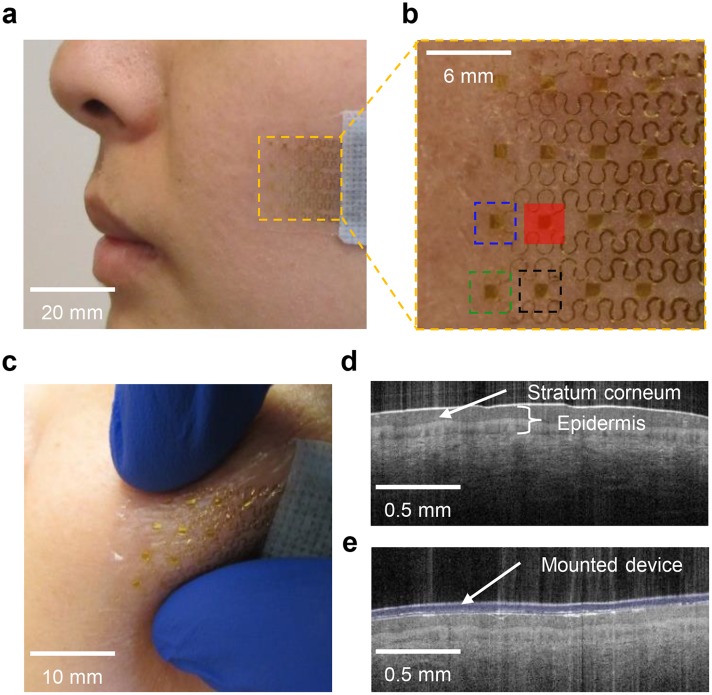
Ultrathin, conformal device for evaluating thermal transport characteristics and validation on human skin. (**a**) Photograph of a device laminated onto a subject’s cheek. (**b**) Magnified view showing the location of the heater (red), a sensing element 3.5 mm away from the heater (blue), 4.7 mm away (black), and 5.8 mm away (green). (**c**) Magnified view during deformation. (**d**) Optical coherence tomography image of a region of a human palm before and (**e**) after mounting the array (blue).

Our recent report [10] introduced a type of thermal sensor with thickness, modulus and thermal mass matched to the epidermis, for spatiotemporal mapping of temperature on the surface of the skin with precision equal to or better than that of state-of-the-art infrared thermography systems. In the present work, advanced versions of this technology enable mapping of not only temperature but also thermal transport properties, including thermal conductivity and thermal diffusivity (and, therefore, the heat capacity per unit volume via the ratio of these two quantities) and their in-plane directional anisotropies. A representative device, shown in Fig. 1a and b mounted on the cheek, consists of a 4×4 array of interconnected filamentary metal structures (Cr/Au; 6/75 nm thick, 10 μm wide) that simultaneously function as thermal sensors and actuators, where the temperature coefficient of resistance of the metal couples changes in temperature to changes in resistance. A thin (<3 μm) film of polyimide encapsulates these structures and their electrical interconnects (Ti/Cu/Ti/Au; 10/500/10/25 nm thick, 50 μm wide) both above and below. A low modulus (35 kPa), thin coating (as small as 5 μm) of a silicone elastomer (Ecoflex 00–30, Smooth-on, USA) provides a conformal, intimate thermal interface directly to the SC. This soft mode of contact, together with the stretchable construction of the overall system, allows for repeated cycles of application, operation and removal without adverse effect on the device or the skin. The maximum heating powers used in experiments reported here introduce readily measurable changes in the temperature at the surface of the skin, but at levels that lie below the human sensory threshold. Optical coherence tomographic (OCT; VivoSight, Michelson Diagnostics, UK) images (Fig. 1c and d) of a region of the skin before and after mounting the device (highlighted in blue) highlight the high level of conformal contact afforded by soft, compliant construction. A wired electrical interface to a USB-powered portable data acquisition system enables operation in non-laboratory settings. See S1 Notes 1–2 and S1–S4 Figs for device fabrication and data acquisition details, and statistical analysis of *in vivo* device temperature readings compared to infrared techniques.

## Results

**Fig 2:**
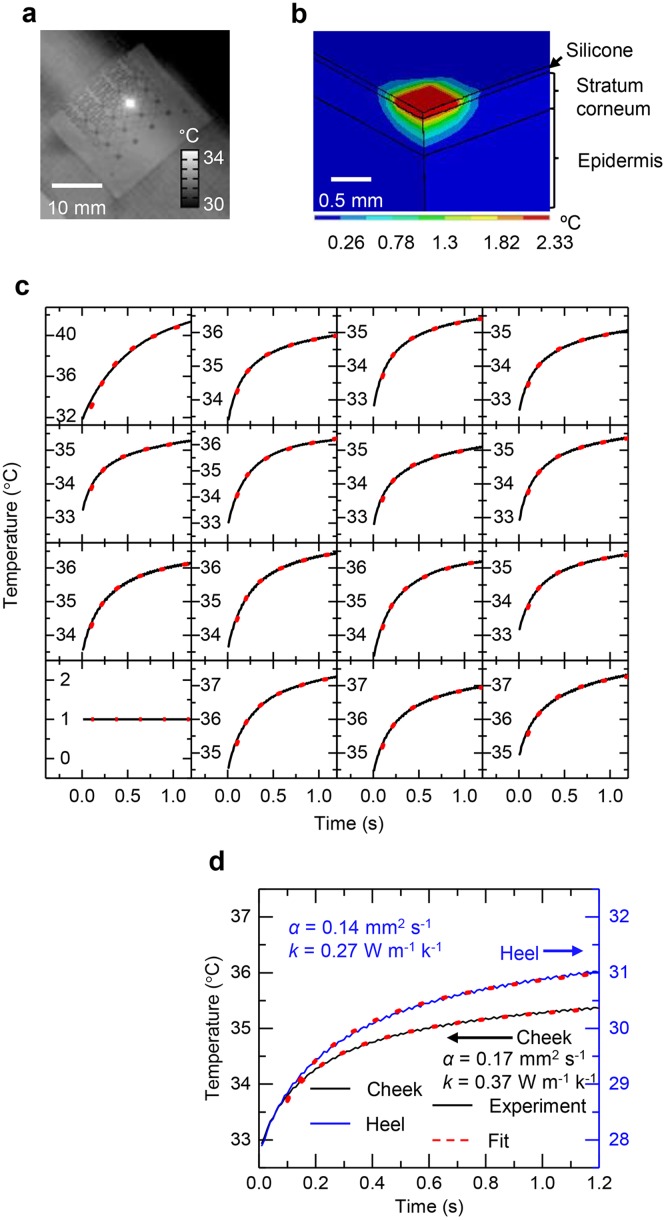
Thermal flow associated with low level transient heating on the surface of the skin. (**a**) Infrared image during heating at a single thermal actuator in an array device on the skin. (**b**) Finite element modelling results for the distribution of temperature during rapid, low level heating at an isolated actuator on the skin, after 1.2 s of heating at a power of 3.7 mW mm^-2^. (**c**) Spatial map of the rise in temperature due to transient heating sequentially in each element in the array. The solid black lines are experimental data; the red dashed lines are best fit calculations. The strong rise shown in upper leftmost element results from local delamination of the device from the skin. (**d**) Experimental data (solid lines) and best fit calculations (dashed lines) for the cheek (black) and heel (blue), along with extracted thermal transport properties.

The sensors and actuators can be used interchangeably in two different modes to assess thermal transport. The first mode uses each element in the array sequentially and independently as both an actuator and a sensor. The measurement occurs quickly (<2 s), with capabilities for spatial mapping. An infrared image collected during the heating sequence (Fig. 2a) shows results of local, rapid heating generated by a single element. Fig. 2b illustrates findings from FEM modeling of the 3-dimensional temperature distribution after 1.2 s of heating, to provide a sense of the depth and lateral spatial scales associated with the measurement. For routine analysis, a simple modification to an analytical treatment [19] in which the heating element is considered as a point heat source in a semi-infinite plane can be valuable. Here,

$$T=T_{\infty }+A_{1}\frac{Q}{2\pi A_{2}k_{skin}}erfc\left(\frac{A_{2}\sqrt{\rho_{skin}c_{p,skin}}}{\sqrt{4k_{skin}t}}\right)$$ (1)

where *T* is the temperature at an effective distance, *A*_*2*_, from the heater, *T*_*∞*_ is the temperature before heating, *Q* is the heating power, *k*_*skin*_ is the thermal conductivity of the skin, *ρ*_*skin*_ *c*_*p*,*skin*_ is the volumetric heat capacity of skin, *t* is time, and *erfc* is the complementary error function. *A*_*1*_ is a parameter that accounts for details associated with the multilayered geometry of the device; its value is calibrated through measurements of materials with known thermal properties similar to those of the skin (water, ethylene glycol and polydimethylsiloxane). *A*_*2*_ accounts for the fact that the thermal actuator (serpentine wire distributed over an area of 1x1 mm^2^) when used as a sensor records a temperature that corresponds to a weighted average over the area of the element. This average temperature, in the model of equation (1), is equivalent to the value at a distance *A*_*2*_ away from an effective point source of heat. As a result, *A*_*2*_ lies between 0 and 0.5 mm, depending on the geometric details and materials properties. In practice, *A*_*2*_ is selected to yield quantitatively accurate results with materials of known thermal properties similar to those of skin. Analysis of *in vivo* data involves an iterative fitting procedure (Matlab, Mathworks, USA) to determine *k*_*skin*_ and the thermal diffusivity (*α*_*skin*_ = *ρc*_*p*,*skin*_ */ k*_*skin*_) using equation (1). Analyses of the sensitivity of the fitting process in the presence of experimental noise indicate maximum uncertainties of 2% and 8% for *k*_*skin*_ and *α*_*skin*_, respectively (S1 Notes 3 and S5 Fig). A similar analysis for errors in sensor calibration indicate maximum uncertainties of 5% and 15%. Measurements described subsequently demonstrate *in vivo* repeatability of better than 6% and 9% for *k*_*skin*_ and *α*_*skin*_ respectively. Comparison of thermal properties determined using equation (1) to those determined using solutions that explicitly integrate numerically over the sensor area indicate discrepancies that lie below the level of these experimental errors (S1 Notes 4).

Examples of representative data (black lines) and calculations based on equation (1) (red dashed lines) for each element across the array appear in Fig. 2c (an example of a malfunctioning sensor that can be quickly identified by the lack of signal, and removed from analysis, is seen in the bottom-most, left-most graph). Fig. 2d presents similar results along with extracted values of *k*_*skin*_ and *α*_*skin*_ for the cheek and the heel pad. The differences between these two cases are significant, and likely result, at least in part, from the variations in the thicknesses of the SC, as described subsequently. The effective depth associated with the measurement can be approximated as [20]

$$\Delta_{p}=\sqrt{\alpha t_{\max}}$$ (2)

where *t*_*max*_ is the characteristic measurement time. This equation gives a probing depth of ~0.5 mm which agrees well with experimental analysis of measurement depth (S1 Notes 5, S6 Fig) as well as the depth of heating shown by the FEM results in Fig. 2b. The depth dependent properties of the skin over this length scale influence the measurements.

**Fig 3:**
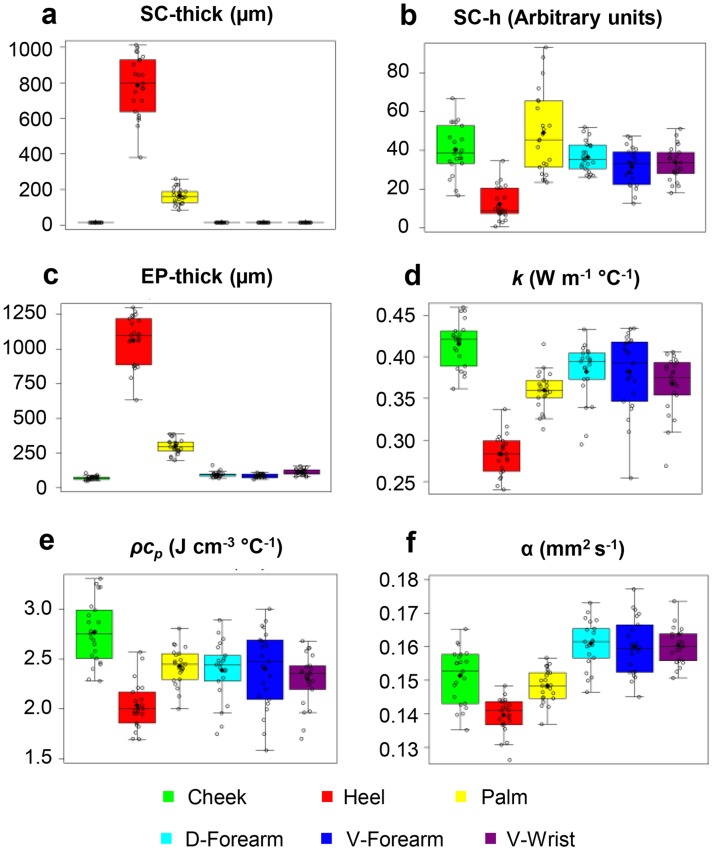
Clinical data distributions. Boxplot representation of the data (open circles). The mean is represented by a black diamond shape. The top and the bottom line of the box are the first and third quartiles, and the middle line of the box is the second quartile—the median. The lower (upper) whisker represents the minimum (maximum) observation above (below) the 1.5 Inter Quartile Range (IQR) below (above) the lower (upper) quartile. Data distributions for the (**a**) stratum corneum thickness (SC-thick), (**b**) stratum corneum hydration (SC-h), (**c**) epidermis thickness (EP-thick), (**d**) thermal conductivity (*k*), (**e**) volumetric heat capacity (*ρc*_*p*_), and (**f**) thermal diffusivity (*α*).

This measurement mode enabled comprehensive, systematic studies of thermal transport characteristics, *in vivo*, on twenty-five human subjects at six different body locations: cheek, dorsal forearm (d-forearm), volar forearm (v-forearm), volar wrist, palm and heel pad. Results for *k*_*skin*_ and *ρ*_*skin*_ *c*_*p*,*skin*_ follow from analysis using equation (1); *α*_*skin*_, which corresponds to their ratio, is useful to consider also, because it determines whether *k*_*skin*_ and *ρ*_*skin*_ *c*_*p*,*skin*_ vary independently across body locations. Correlations between skin thermal properties to SC hydration measured using a corneometer (Cutometer MPA 580, Courage + Khazaka Electronics GmbH), EP thickness and SC thickness measured using OCT provide further insights into the results. Fig. 3, which shows the distribution of these variables using a boxplot representation, reveals three distinct clusters for the thermal parameters: 1 cheek; 2 heel; and 3 palm, wrist, v-forearm and possibly d-forearm (the spread in the data here is relatively large due to the interference of hair on the measurement). Some separation occurs between the palm and the wrist/v-forearm/d-forearm, but to a degree that is not apparent from the univariate descriptive analysis. OCT yielded accurate values of SC thickness for the palm and heel pad but not for the follicular regions, where previous studies indicate a typical value of ~15 μm [21–23].

**Fig 4:**
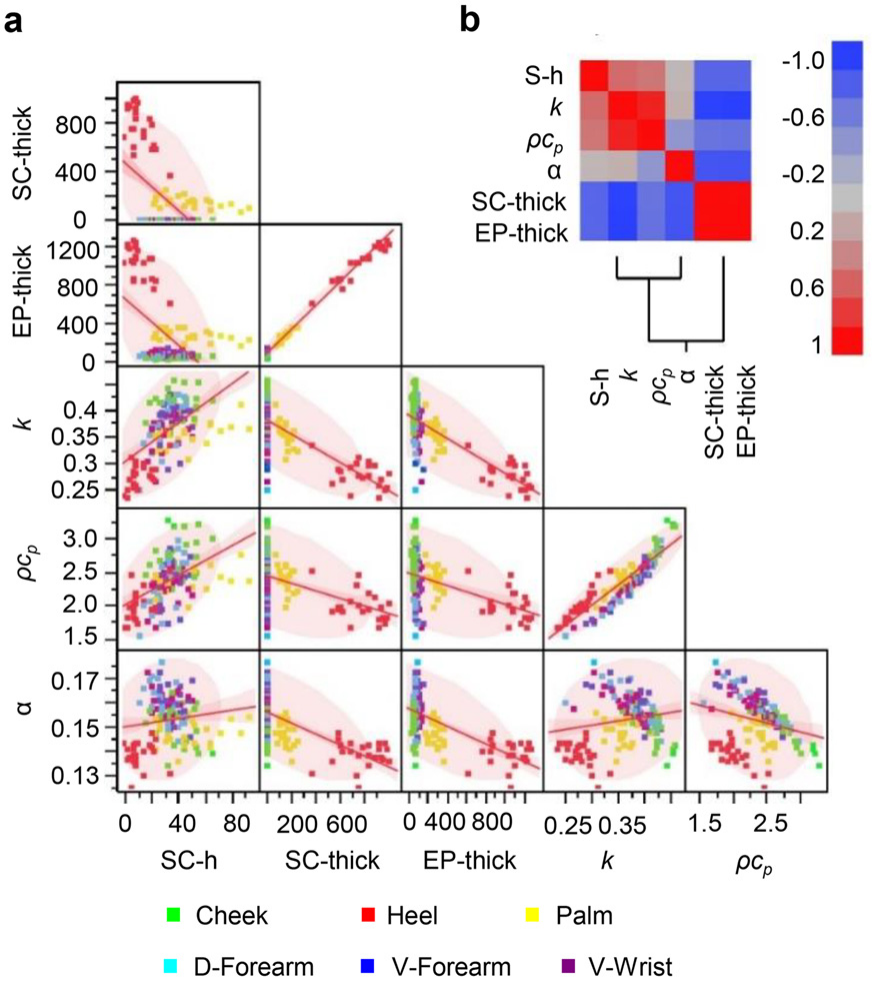
Clinical data correlation analysis. (**a**) Scatterplot matrix representation for the entire data set (all 6 body locations: cheek, volar and dorsal forearm, wrist, palm, and heel on 25 total subjects). Pairwise correlation analyses include the thermal characteristics (*k*, W m^-1^°C^-1^; *ρc*_*p*_, J cm^-3^°C^-1^; *α*, mm^2^ s^-1^) and stratum corneum thickness (SC-thick, μm), epidermal thickness (EP-thick, μm), and stratum corneum hydration (SC-h, arbitrary units). Data for different body areas are represented by different colors. The red line represents the pairwise linear regression slope. The pink shaded clouds represent the 95% bivariate normal density ellipse. Assuming the variables are bivariate normally distributed, this ellipse encloses approximately 95% of the points. (**b**) The bivariate correlations for the entire data set are represented using a color coding (HeatMap) scheme associated with a clustering of the descriptors. Dark red is associated with Pearson Correlation Coefficient, R, equal to 1 and dark blue is associated to R = -1. The Pearson correlation coefficients are given in S1 Table.

**Fig 5:**
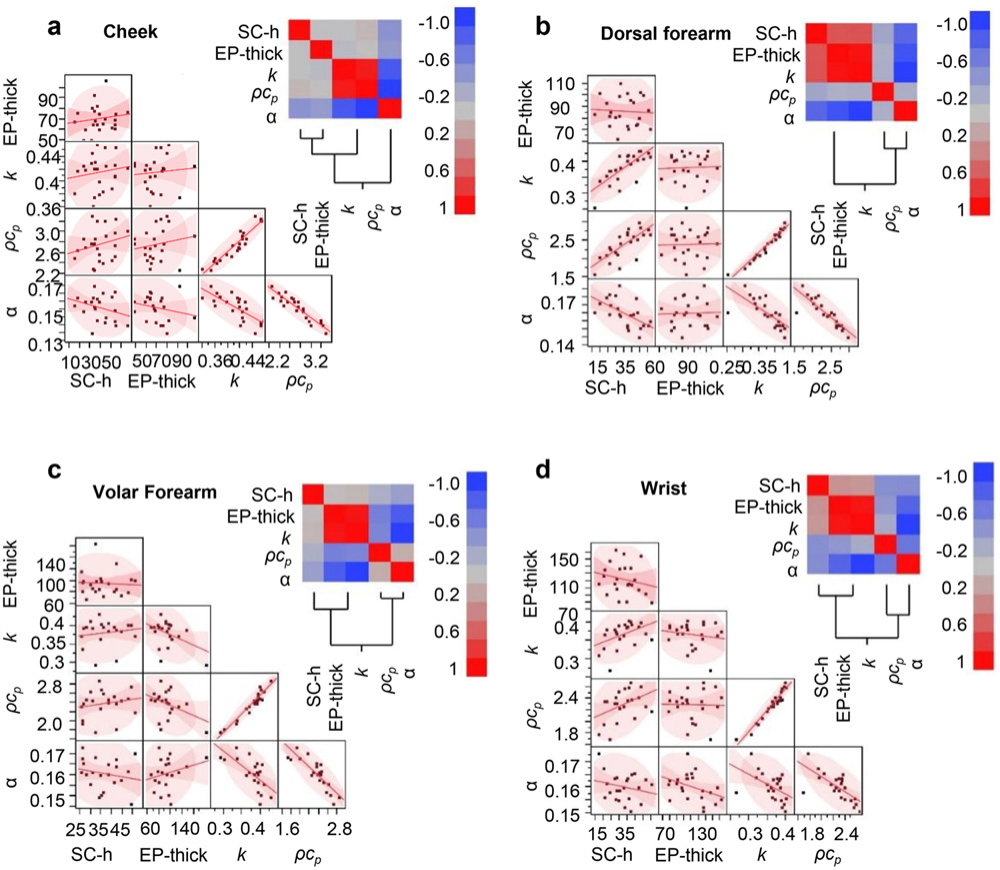
Clinical data correlation analysis for regions without significant stratum corneum thickness. The same correlation analysis as in Fig. 6 for the (**a**) cheek, (**b**) dorsal forearm, (**c**) volar forearm and (**d**) wrist.

**Fig 6:**
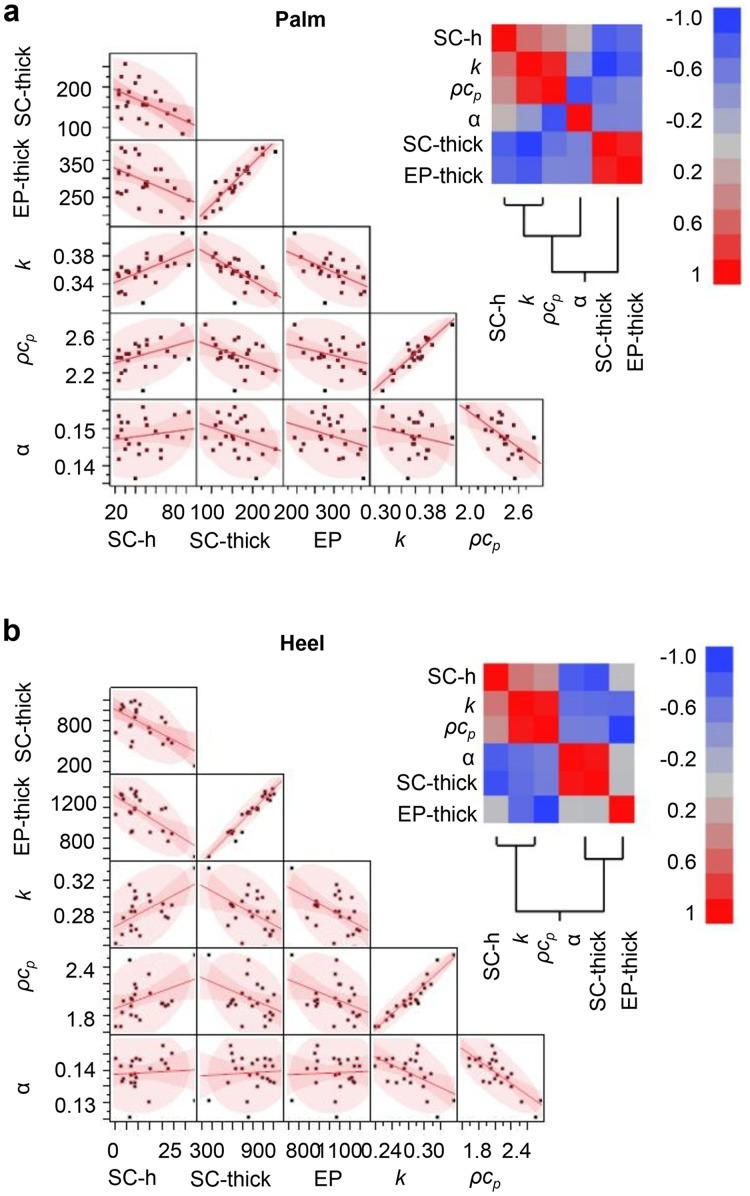
Clinical data correlation analysis for regions with significant stratum corneum thickness. The same correlation analysis as in Fig. 6 for the (**a**) palm and (**b**) heel.

Pairwise correlation analyses for the skin thermal parameters, SC and EP thickness, and SC hydration appear in Fig. 4 for the entire data set, in Fig. 5 for each follicular region and in Fig. 6 for the palm and heel pad. The data show strong positive correlation between SC hydration and *k*_*skin*_ and *ρ*_*skin*_ *c*_*p*,*skin*_. The ratio *α*_*skin*_ exhibits a positive, but weaker, correlation with SC hydration. The data also indicate a strong negative correlation between SC/EP thickness and all three thermal properties (*k*_*skin*_, *ρ*_*skin*_ *c*_*p*,*skin*_ and *α*_*skin*_). The EP thickness correlates with the SC thickness. SC is a significant fraction of the EP, especially in palmar regions, i.e. palm and heel pad. The SC thickness and SC hydration of the palmar regions show negative correlation. The strength of correlation depends strongly on body location (Figs. 5 and 6, and S1 Table).

**Fig 7:**
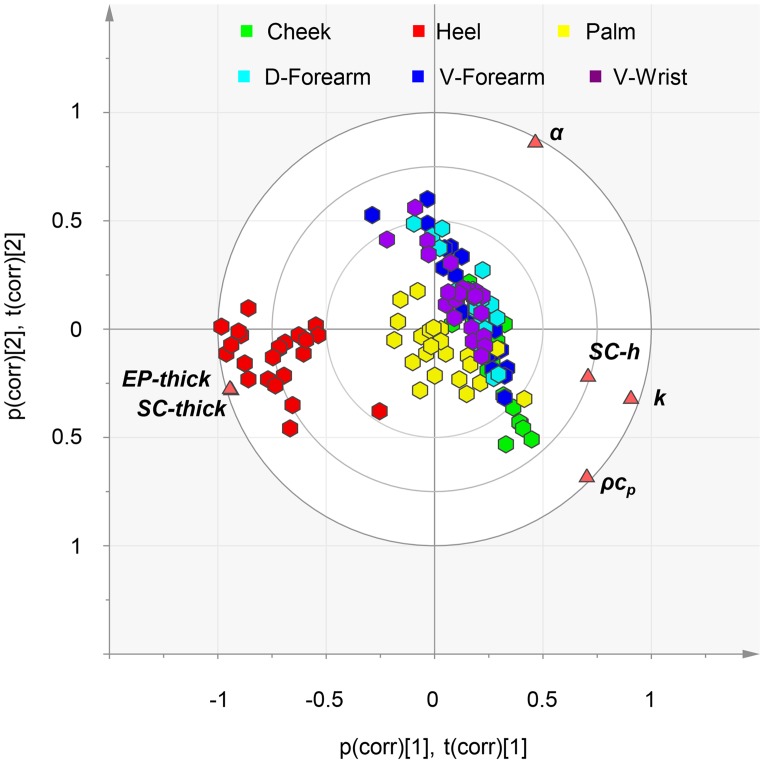
Principal Component Analysis. Global, multivariate correlation analysis. On the Biplot each body location is represented by polygons and the descriptors by triangles.

Principal component analysis (PCA), as a global multivariate approach of correlation analysis, appears in Fig. 7 and S8 Fig. PCA offers a graphical representation of both individuals and descriptors, with an ability to reveal hidden patterns in the data. The eigenvalues show that the first PCA axis explains 71% of the variance. The second and third components correspond to 20% and 7%, respectively. Hence, three components explain 97% of the inertia. In the biplot representation, the data, by location, are represented using scores coordinates, where the scores are the Principal Components (PCs). The first PC mainly separates observations of the heel from the other body areas (Fig. 7 and S8a Fig). Discrimination also occurs, to a lesser extent, between the cheek and a group composed of palm, v-forearm, d-forearm and wrist (S8a Fig). The second PC discriminates the arm and wrist location from the others (S8b Fig). The third PC differentiates the palm (S8c Fig). Based on the PCs, four distinct clusters occur within the data set: heel, cheek, palm, and wrist/v-forearm/d-forearm indicating four distinct locations with different physical properties. Descriptors close together on the biplot are highly correlated and conversely descriptors opposed are highly anti-correlated. On the biplot, SC hydration, thermal conductivity and volumetric heat capacity form one group and EP thickness and SC thickness for another with the two groups opposed on the first axis. This conveys the strong positive correlation of descriptors from the same group and conversely the negative correlation of descriptors from different groups. Interestingly, the thermal diffusivity is more linked to the second axis, and therefore quite independent to the other descriptors. This is consistent with previous remarks based on Pearson correlation coefficients.

**Fig 8:**
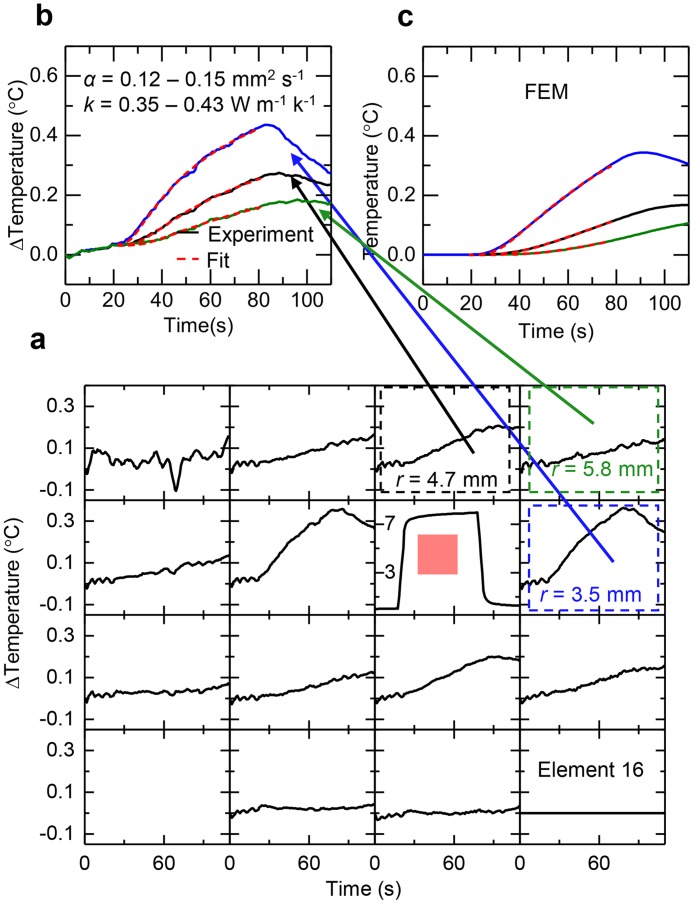
Spatial mapping of thermal transport associated with low level heating on the surface of the skin. (**a**) Spatial map of the changes in temperature at each sensor (i.e. element) in the array. The data processing uses an adjacent-average filter (window size = 8 s) and normalization to Element 16. The red highlight and colored boxes represent the elements boxed in the same colors in Fig. 1b. (**b**) Change in temperature at elements 3.5 mm away (blue), 4.7 mm away (black) and 5.8 mm away (red) from element responsible for thermal actuation. The solid and dashed lines represent experimental data and best fit calculations, with *k* ~ 0.35–0.43 W m^-1^ K^-1^ and *α* ~ 0.12–0.15 mm^2^ s^-1^. (**c**) Results of finite element modelling of an array on a cheek, in the same arrangement as **b**.

**Fig 9:**
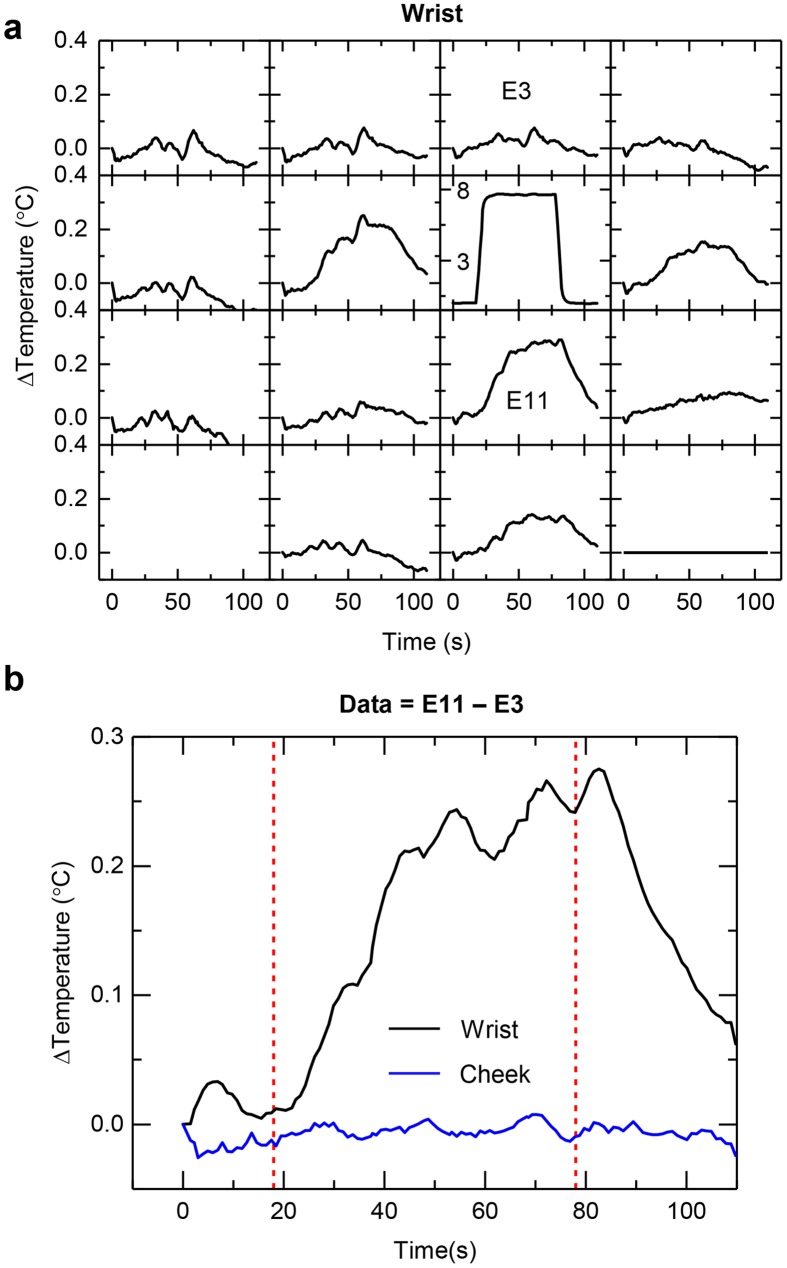
Anisotropic convective effects associated with near surface blood flow. (**a**) Spatial map of changes in temperature at each element for a device located at the volar aspect of the wrist. The position of the thermal actuator coincides with a large vein. (**b**) Difference in temperature between element 11 (E11) and element 3 (E3). The results show effects of anisotropic heat flow in the wrist, compared to isotropic distributions typically observed on a region of the body such as the cheek. The vertical red dashed lines correspond to initiation and termination of heating, respectively.

In addition to intrinsic properties of the skin itself, a second mode for characterizing thermal transport allows investigation of directional anisotropies and other effects related, for example, to blood flow near surface arteries and veins. Here, application of electrical power (8 mW / mm^2^ for 60 s) to a selected element (highlighted by the red box in Fig. 2b (optical image) and Fig. 8a (data)) introduces a controlled level of heating while the temperature of this element and all others in the array are simultaneously recorded as a function of time. Processing the data with an adjacent-averaging filter (window size = 8 s), and subtracting the response of the sensor furthest from the actuator (Element 16) from that of each of the other sensors in the array minimizes effects of fluctuations in the ambient temperature. Here, the actuator can be approximated as a point source of heat, such that the transient temperature profile at a distance *r* can be written

$$T=T_{\infty }+A_{1}\frac{Q}{2\pi r(t)k_{skin}}erfc\left(\frac{r(t)\sqrt{\rho_{skin}c_{p,skin}}}{\sqrt{4k_{skin}t}}\right)$$ (3)

where *T*_∞_ is the temperature before heating, *Q* is the heating power, *k*_*skin*_ is the thermal conductivity of the skin, *ρ*_*skin*_ *c*_*p*,*skin*_ is the volumetric heat capacity of skin, *t* is time, and *erfc* is the complementary error function. *A*_*1*_ is a parameter that accounts for details associated with the multilayered geometry of the device; its value is calibrated through measurements of materials with known thermal properties similar to those of the skin (water, ethylene glycol and polydimethylsiloxane). *r(t)* represents the effective distance of the sensor from the heating element and takes the form of a time dependent function that accounts for the finite spatial area of the sensing element (S1 Notes 6). *k*_*skin*_ and *α*_*skin*_ can be determined in a iterative process similar to that used in equation (1). The treatment of *r* causes a maximum relative error of <2% in the determination of *k*_*skin*_ and *α*_*skin*_ compared to those values determined by integrating equation (3) over its area at each time point (S1 Notes 6). Representative results for different sensors appear in Fig. 8b. Finite element modeling (FEM) of the full device construct on a bilayer model of the skin yields temperature profiles (Fig. 8c) that closely match those observed in experiment. This measurement configuration provides additional information beyond that determined in equation (1) in the form of anisotropy in heat transport, at the expense of precision in the determination of thermal properties. Fig. 8 is an example of a skin area where the heat transport is strongly isotropic, while Fig. 9 illustrates the spatial changes in thermal transport on an area of skin with a significant anisotropic component to heat transport. Convective effects associated with blood that flows through vessels near the skin surface can induce in-plane, directional anisotropies in heat transport characteristics. Fig. 9 illustrates the effect when a device mounted on the volar aspect of the wrist includes a thermal actuator located over a near surface vein. The spatiotemporal temperature map in Fig. 9a shows a significantly larger increase in temperature at the sensor located downstream (more proximal to the body, labeled E11) from the actuator, compared the one upstream (more distal to the body, labeled E3), relative to the direction of blood flow. Fig. 9b highlights one method to quantitatively assess the anisotropy in thermal flow. Here, the response of sensor E3 is subtracted from that of sensor E11 (sensors E3 and E11 are equidistant from the heating element, arranged on opposing sides of the heater) for the case on the wrist, which shows strong anisotropy due to blood flow, and for the case of isotropic data from a representative case on the cheek. The degree of anisotropic transport varies in strength over the twenty-five subjects due to differences in the locations and sizes of blood vessels and their associated flow properties. Such measurement capabilities have relevance in the determination of cardiovascular health, through inferred measurements of blood flow, both naturally and in response to stimuli such as temporary occlusion.

## Discussion

In summary, the work reported here reveals intrinsic thermal transport properties of the skin, including relationships to vascularization, blood flow, stratum corneum thickness and hydration level, made possible by expanded capabilities in soft ultrathin, non-invasive measurement systems that offer clear advantages compared to traditional approaches. As a demonstration of the new, *in vivo* measurement capabilities enabled by the device presented here, a clear relationship between skin hydration and *in vivo* thermal transport properties is shown across six body locations on twenty-five subjects. The data also reveal that the *in vivo* thermal transport properties of skin are not uniquely a function of hydration, but are also influenced by the structural makeup of the skin, as well as local blood flow characteristics. Obtaining similar data with alternative measurement techniques would require either expensive and complex optical thermography and laser heating systems, or bulky single-point probes that can have undesired effects on the skin properties of interest due the pressure that must be applied to skin when measured *in vivo*. The device and data presented here provide a foundational step for a new approach to the measurement of *in vivo* skin thermal properties, as well as new statistical data about the correlations between skin thermal transport properties, and skin hydration and structural makeup. Immediate further opportunities include use in studies of dermatological diseases, such as melanoma, rosacea and hyperpigmentation and their progression over time. The same techniques also offer ability to examine the effectiveness of dermatologically active compounds. Developments in wireless technology will provide a path to continuous monitoring of skin properties and function using these concepts.

## Methods

### Fabrication of Epidermal Thermal Sensing Array

Fabrication begins with a 3” Si wafer coated with a 200 nm layer of poly(methyl methacrylate), followed by 1 μm of polyimide. Photolithographic patterning of a bilayer of Cr (6 nm)/Au (75 nm) deposited by electron beam evaporation defines the sensing/heating elements. A second multilayer of Ti (10 nm)/Cu (500 nm)/Ti (10 nm)/Au (25 nm), lithographically patterned, forms the connections to sensing/heating elements and non-oxidizing bonding locations for external electrical connection. A second layer of polyimide (1 μm) places the sensing/heating elements in the neutral mechanical plane and provides electrical insulation and mechanical strain isolation. Reactive ion etching of the polyimide defines the mesh layout of the array and exposes the bonding locations. A water-soluble tape (5414, 3M, USA) enables removal of the mesh layout from the Si wafer, to expose its back surface for deposition of Ti (3 nm)/SiO_2_ (30 nm) by electron beam evaporation. Transfer to a thin silicone layer (5 μm; Ecoflex, Smooth-On, USA) spin-cast onto a glass slide, surface treated to reduce adhesion of the silicone, results in the formation of strong bonds due to condensation reactions between exposed hydroxyl groups on and the SiO_2_ and silicone. Immersion in warm water allows removal of the tape. A thin (100 μm), flexible, conductive cable (HST-9805–210; Elform, USA) bonded with heat and pressure to contacting pads at the periphery serves as a connection to external electronics. A final layer of silicone (70 μm) in combination with a frame of medical tape (Ease Release, 3M, USA) provides sufficient mechanical support to allow repeated (hundreds of times) use of a single device.

### Data Acquisition for Epidermal Thermal Sensing Array

The epidermal thermal sensing array is connected to external data acquisition electronics via a thin (100 μm) silver ink/polymer composite cable (HST-9805–210; Elform, USA). Resistance and voltage values across sensor/actuator elements are recorded by a USB-powered digital multimeter (USB-4065; National Instruments, USA). In order to heat elements, controlled current is supplied by a DC current source (6220 DC Current Source; Keithley, USA). The temperature during heating is monitored by recording the voltage across the heating element while receiving constant current input. The sensors are time-multiplexed via a USB-powered multiplexing circuit (U802; Ledgestone Technologies, USA).

### Experiments on Human Subjects

The volunteers consisted of healthy females, age between 18 and 45 years old, with healthy, intact skin of type II–IV according to the Fitzpatrick classification, recruited by Stephens & Associates, TX, USA. Approval by Stephens & Associates IRB: Protocol No. C14-D100 (ACR/TEMP/1416). Subjects provided written consent. The six investigational areas included the cheek, volar forearm, dorsal forearm, volar wrist, palm, and heel. Each subject acclimated to room temperture for 15 min immediately prior to measurement. The investigational areas were then gently cleaned with isopropyl alcohol, water, and dried with a swab to avoid skin irritation. Pictures were taken before and after the experimental procedures. SC hydration measurements used a 3 Cutometer MPA 580 (Courage + Khazaka Electronics GmbH). Skin temperature was evaluated using a handheld IR thermometer (DermaTemp, Exergen Co., USA). Calibration of the experimental measurement system introduced here occurred at a single temperature point (room temperature). Evaluations involved lamination of the device onto the investigational area, collection of relevant data, followed by removal. Three additional corneometer readings were then collected, followed by measurements by optical coherence thomograpy (VivoSight,Michelson Diagnostics, UK). The individual pictured in Fig. 1 has given written informed consent (as outlined in the PLOS consent form) to publish these case details.

### Statistical Analyses

Box plot representations (SAS statistical software release 9.3, SAS Institute Inc., Cary, NC, USA) illustrate variables and trends by body location. The pairwise Pearson correlation coefficients were displayed as tables, scatterplot matrices, or heat map representations using JMP statistical software release 10.0 (JMP is a trademark of SAS Institute). Principal Component Analysis serve as a global multivariate approach with a biplot representation of individuals and descriptors (SIMCA statistical software release 13.0, UMETRICS, Umeå, Sweden).

## Supporting Information

S1 Notes Supplementary Notes 1–6: Supporting text, figures and tables. (PDF)

S1 Fig Device construction and temperature comparison to IR measurements. (**a**) Optical image of 4x4 thermal sensing array, showing the bonding location of the thin, flexible cable (ACF connection). (**b**) Magnified image of a single sensor/actuator element, showing the 10 μm wide, serpentine configuration. (**c**) Cross-sectional schematic showing the device layout on skin. (**d**) Comparison of temperature device readings on six body locations on each of twenty-five subjects, as compared to IR measurements. Pearson correlation coefficient = 0.98.
(TIF)

S2 Fig Representative photographs of each body location before, during, and after measurements. Images show each body location before application of the thermal sensing array, with the device applied to skin during heating applications for thermal measurements, and then after device removal. No irritation is observed as a result of heating, or wearing the device. Body locations are (**a**) cheek, (**b**) volar forearm, (**c**) dorsal forearm, (**d**) wrist, (**e**) palm, and (**f**) heel.
(TIF)

S3 Fig Temperature variations across body locations. (**a**) Variation in temperature data between different subjects on different body locations for thermal sensing array (blue) and IR thermometer (red). (**b**) Inter- and intra-subject variance for the thermal sensing array and IR thermometer.
(TIF)

S4 Fig Temperature variations across body locations for each subject. Variation in temperature data between different subjects on different body locations for thermal sensing array (blue) and IR thermometer (red).
(TIF)

S5 Fig Analysis of fitting process sensitivity with experimental error. (**a**) Experimental precision fitting error analysis of representative *in vivo* data on a human heel. Experimental error range is given by 3x the standard deviation of temperature readings from the mean. (**b**) Experimental accuracy fitting error analysis of representative *in vivo* data on a human heel and (**c**) a human cheek. Experimental error range is given by the 95% confidence interval of temperature readings due to calibration errors.
(TIF)

S6 Fig Experimental determination of measurement probing depth. Measured thermal conductivities by the thermal sensing array for different thickness of a silicone with thermal properties similar to skin (Sylgard 170, Dow Corning, USA; *k* = 0.39 W m^-1^ K^-1^, *ρ* = 1370 kg m^-3^) on copper. The measured thermal conductivity rises rapidly when the silicone layer becomes thinner than the probing depth, which is given by Eq. 2 to be approximately 0.5 mm.
(TIF)

S7 Fig Solutions for *r(t)*. Numerically determined solutions for *r(t)* over the appropriate measurement time, determined using *k* = 0.35 W m^-1^ K^-1^ and *α* = 0.15 mm^2^ s^-1^, for (**a**) *r* = ~3.5 mm, (**b**) *r* = ~4.7 mm, and (**c**) *r* = ~5.8 mm. (**d**) Example temperature rise solutions for a sensor ~3.5 mm away using the integrated solution of Eq. **S5**, *r(t)* given in **a** with Eq. **S6**, and various time independent values of *r* with Eq. **S6**. *r(t)* gives the smallest discrepancy with Eq. **S5** at <1%, and time independent average values of *r* give discrepancies <5%.
(TIF)

S8 Fig Principle component analysis. Boxplot representation of principal components by body location, and their corresponding relation to measured parameters. (**a**) Box plots and correlation weights of the first principal component, (**b**) the second principal component and (**c**) the third principal component.
(TIF)

S1 Table Supplementary Table Pearson Correlation coefficients for the correlation analyses (Figs. 4–6). (TIF)
