# Supplementary material for: Thermal Transport Characteristics of Human Skin Measured In Vivo Using Ultrathin Conformal Arrays of Thermal Sensors and Actuators
Source: PLoS One. 2015 Feb 6;10(2):e0118131. doi: 10.1371/journal.pone.0118131 (PMC4319855; doi:10.1371/journal.pone.0118131)
Supplement: S1 Notes — (PDF) [file pone.0118131.s001.pdf]

Supplementary Information for:

Thermal Transport Characteristics of Human Skin Measured In Vivo Using Ultrathin Conformal Arrays of Thermal Sensors and Actuators

R. Chad Webb[1,†], Rafal M. Pielak[2,†], Philippe Bastien[3], Joshua Ayers[1], Juha Niittynen[4], Jonas Kurniawan[1], Megan Manco[5], Athena Lin[1], Nam Heon Cho[1], Viktor Malyrchuk[1], Guive Balooch[2,6,*] and John A. Rogers[1,*]

[1]*Frederick Seitz Materials Research Laboratory, Department of Materials Science and Engineering, University of Illinois at Urbana-Champaign, Urbana, IL 61801, USA*

[2]*L'Oréal California Research Center, 953 Indiana St. San Francisco, CA 94107*

[3]*L'Oréal Research and Innovation, 1 av. Eugène Schuller, 39601 Aulnay sous Bois, France*

[4]*Tampere University of Technology, Department of Electronics and Communication Engineering, Korkeakoulunkatu 3, P.O. Box 692, FI-33101 Tampere, Finland*

[5]*L'Oréal Early Clinical, 133 Terminal Ave. Clark, NJ 07066*

[6]*L'Oréal Digital Incubator, 133 Terminal Ave. Clark, NJ 07066*

[†]These authors contributed equally to this work

[*]To whom correspondence should be addressed. Guive Balooch: gbalooch@rd.us.loreal.com, John A. Rogers: jrogers@illinois.edu

Supplementary Note 1: Fabrication procedure for ultrathin thermal sensing arrays

Prepare polymer base layers

1. Clean a 3” Si wafer (Acetone, IPA -> Dry 5 min at 110 °C).
2. Spin coat with PMMA (poly(methyl methacrylate) 495 A2 (Microchem), spun at 3,000 rpm for 30 s.
3. Anneal at 180 °C for 1 min.
4. Spin coat with polyimide (PI, poly(pyromellitic dianhydride-co-4,4′ -oxydianiline), amic acid solution, Sigma-Aldrich, spun at 4,000 rpm for 30 s).
5. Anneal at 110 °C for 30 s.
6. Anneal at 150 °C for 5 min.
7. Anneal at 250 °C under vacuum for 1 hr.

Deposit first metallization

8. E-beam 6/75 nm Cr/Au.
9. Pattern photoresist (PR; Clariant AZ5214, 3000 rpm, 30s) with 365 nm optical lithography through iron oxide mask (Karl Suss MJB3).
   Develop in aqueous base developer (MIF 327).
10. Etch Au with TFA Au etchant (Transene).
11. Etch Cr with CR-7 Cr Mask Etchant (Cyantek).
12. Remove PR w/ Acetone, IPA rinse.
13. Dry 5 min at 150 °C.

Deposit second metallization

14. E-beam 10/500/10/25 nm Ti/Cu/Ti/Au.
15. Pattern PR AZ5214.
16. Etch Au with TFA Au etchant.
17. Etch Ti with 6:1 Buffered Oxide Etchant.
18. Etch Cu with CE-100 etchant (Transene).
19. Etch Ti with 6:1 Buffered Oxide Etchant.
20. Remove PR w/ Acetone, IPA rinse.
21. Dry 5 min at 150 °C.

Isolate entire device

22. Spin coat with PI.
23. Anneal at 110 °C for 30 s.
24. Anneal at 150 °C for 5 min.
25. Anneal at 250 °C under vacuum for 1 hr.
26. Pattern photoresist (PR; Clariant AZ4620, 3000 rpm, 30s) with 365 nm optical lithography through iron oxide mask (Karl Suss MJB3).

Develop in aqueous base developer (AZ 400K diluted 1:3, AZ 400K:Water).

27. RIE (150 mTorr, 20 sccm O2, 200 W, 20 min).

Release and transfer

28. Release w/ boiling Acetone.
29. Transfer to water-soluble tape (Wave Solder Tape, 5414, 3M).
30. E-beam 3/30 nm Ti/SiO2.
31. Transfer to ~10 μm silicone sheet (Ecoflex, Smooth-on Co.) coated on silanized glass slide.
32. Immerse in warm water to dissolve tape.
33. Immerse quickly in Chrome Mask Etchant to remove any remaining residue.
34. Bond thin, flexible cable (Elform, HST-9805-210) using hot iron with firm pressure.
35. Apply additional silicone (10-100 um) by doctor blade
36. Apply silicone medical tape frame (Ease Release Tape, 3M).
37. Remove device.

In order to provide a more appropriate system for repeated clinical use, we improve upon our initially demonstrated system in several ways. First, an electron beam evaporated metallic stack of Ti/Cu/Ti/Au (10/500/10/25 nm) replaces the expensive Au interconnect wiring system. This system provides the desired low resistivity interconnects while using minimal Au as a contact material. Narrow line widths (10 μm) in the sensing/heating elements provide high resistance in a small spatial area, shown in Fig. S1**b**, minimizing undesired heating in interconnect wires. A thin layer of Ecoflex (smooth-on, ETC) polymer between the sensor/heater elements (Fig. S1**c**) and the skin improves the adhesion directly between the heating element and the skin, minimizing errors in thermal transients that may be caused by air gaps. Finally, a silicone adhesive based tape (Ease Release, 3M, USA) functions as a frame for the device, providing a flexible but robust mechanical support for repeated use over >100 applications (see Fig. S2 for images before, during, and after measurement on each body location in the clinical study). Finally, the data acquisition and control system is in the form of a low cost, USB-powered portable system for practical clinical use. High temperature resolution is achieved by the 22-bit digital multimeter (USB-4065, National Instruments, USA) and time-multiplexing is achieved by the use of a USB-powered, voltage isolated switch circuit (U802, Ledgestone Technologies LLC, USA).

Supplementary Note 2: Temperature measurements across all body locations

In order to verify temperature accuracy, temperature recordings by the device array are compared to recordings by a commercial infrared thermometer (DermaTemp, Exergen Co., USA) on each body location (Fig. S1**d**). The temperature values correlate well (Pearson's correlation coefficient, R, = 0.98, slope = 0.95 ± 0.02, intercept = 2.5 ± 0.5, standard errors), verifying the value of the device in the context of epidermal temperature

sensing across varied body locations, as demonstrated previously [1]. Average temperature variations between body locations are shown in Fig. S3, and temperature variations of measured on each body location on each subject are shown in Fig. S4.

Supplemental Note 3: Estimated error in fitting models for clinical study

The fitting model described by equation (1) and Fig. 2 is used to determine thermal property data for the 150 body locations measured during the clinical study. In this fitting procedure, two parameters, thermal conductivity and thermal diffusivity, are fit simultaneously. We assess the potential error in this fitting procedure by fixing one of the parameters, and allowing the other to float to determine the best fit with experimental data. In order to determine the fixed parameter value, we initially conduct the fit with both parameters floating to determine the best fit with experimental data (Fig. S5, red dashed line). We then fix one parameter, with a relative error from the best fit value, and allow the second parameter to float to determine a new best fit. We increase the error introduced to the fixed parameter until the new best fit curve falls just outside the error range of the experimental data (Fig. S5; best fit curves after applying error shown as blue and green dashed line; error range of experimental data shaded in red). The error range associated with the precision (i.e. the sensitivity of measurements using the same device one measurement to the next) of experimental data (Fig. S4**a**) is given as ±0.04 °C, which is >3σ, where σ = 0.013°C is the *in vivo* experimental standard deviation of error from the mean. This error analysis conducted on several sets of *in vivo* data from our clinical study results in 2-3% potential error in the value of k and 8% potential error in the value of α, with representative analyses from the heel shown in Figs. S5**a**. Each *in vivo* measurement involves solutions to k and α from each of fifteen sensors in the array. The average standard deviation across all body locations, excluding the dorsal forearm which has large deviations due to hair on some subjects, of all subjects is 6% (0.02 W m^{-1} K^{-1}) and 9% (0.013 mm^2 s^{-1}) for k and α respectively.

The error range associated with the sensor accuracy (i.e. the reliability of measurements when using different devices on measurement to the next) of experimental data is given by the 95% confidence interval of the sensor calibration of temperature sensitivity. This error analysis conducted on several sets of *in vivo* data from our clinical study results in 4-5% potential error in the value of k and 15% potential error in the value of α, with representative analyses from the heel and cheek shown in Figs. S5**b** and S5**c** respectively.

Supplemental Note 4: Error analysis of equation (1) approximations

The algorithm used to calculate skin thermal transport properties from transient heating in individual elements, shown in equation (1), is a convenient approximation to the solution of the average temperature of a small square with finite dimensions during transient heating. The approximation in equation (1) assumes that the average

temperature in the square can be approximated by assuming a point heat source at the center of the square, and a temperature rise some distance A_2 away from the point source. The iteration of equation (1) is computationally inexpensive, which allows for rapid computation of the data from each element in the array. The potential error associated with equation (1) is investigated by comparison to the more exact, and computationally expensive, solution given by Gustafsson [2]

$$\overline{\Delta T(\tau)} = \frac{P_0 H(\tau)}{4\pi^{\frac{1}{2}} bk} \tag{S1}$$

where P_0 is the power output of the heater, b is the half width of the square heating element (0.5 mm for the our device), k is the thermal conductivity,

$$\tau = \frac{t\alpha}{b^2} \tag{S2}$$

where α is the thermal diffusivity, t is time and

$$H(\tau) = \int_0^{\tau} dv \{\mathrm{erf}(v^{-1}) - \pi^{-1/2} v[1 - exp(-v^{-2})]\}^2 \tag{S3}$$

where *erf* is the error function given by

$$\mathrm{erf}(x) = 2\pi^{-1/2} \int_0^{x} dv \exp(-v^2)\,. \tag{S4}$$

equation (S1) accounts for the finite spatial extent of the heater to determine the average measured temperature of the heater. However, iterating this solutions of equations (S1) – (S4) over the large body of data with the high frequency measurement of data across many elements in an array quickly becomes computationally intensive. In order to compare the error using equation (1), we compare the thermal properties, k and α, determined on a representative dataset using equation (1) to those determined by the iteration procedure of equations (S1) – (S4), once calibrated with known calibration media (water and ethylene glycol). The average discrepancy between the two procedures in the solution for k and α is 3% and 8%, respectively, which is within the previously described error ranges due to noise. These potential errors will manifest in the form of constant accuracy offset that will be consistent across all devices. As a result, these potential errors will not influence the precision between measurements, different devices or the resultant correlation statistics that of primary interest.

Supplemental Note 5: Estimation of measurement depth

The measurement technique outlined by equation (1) results in thermal property values that are a weighted average of the values encountered through the depth of skin that is probed by the measurement. The measurement depth can be approximated by equation

(2), which results in a measurement depth of ~500-1000 μm in skin. We verify this result experimentally by conducting measurements on varying thickness of a polymer, with thermal properties similar to skin (Sylgard 170, Dow Corning, USA), on a base substrate of copper. The copper acts a thermal ground plane that will result in rapidly increasing measured thermal properties as the measurement depth approaches the polymer thickness. The resultant measured thermal conductivities on various thicknesses of polymer on copper are shown in Fig. S6, and the measured thermal conductivities begin to rise rapidly at a polymer thickness of approximately 500 μm.

Supplemental Note 6: Error analysis of equation (3) approximations

The measurement configuration outlined by equation (3) and Fig. 8 assumes a discrete distance, *r*, away from a point source heater. The sensors in the array in use here have a finite aerial spatial extent of 1 mm x 1 mm, with <3 μm thickness. The temperature increase recorded by a sensor corresponds to the average temperature increase over the sensor area. Assuming isotropic radial conduction, valid for cases without anisotropic convective transport due to blood, the average temperature across the sensor, $\bar{T}$, is approximately equal to the average temperature rise between points r_1 and r_2 away from a point source heater, given by

$$\bar{T} = \frac{\int_{r_1}^{r_2} \frac{Q}{2\pi r k_{skin}} erfc\left(\frac{r\sqrt{\rho_{skin} c_{p,skin}}}{\sqrt{4k_{skin}t}}\right) dr}{r_2 - r_1} \tag{S5}$$

where r_1 and r_2 are 1 mm apart and represent the distances of the sensor near and far edges, respectively, from the heater. equation (S5) can be approximated by

$$\bar{T} = \frac{Q}{2\pi r(t) k_{skin}} erfc\left(\frac{r(t)\sqrt{\rho_{skin} c_{p,skin}}}{\sqrt{4k_{skin}t}}\right) \tag{S6}$$

where the integral average over the sensor in equation (S5) has been replaced by *r(t)*, a time dependent characteristic distance. *r(t)* is determined numerically by setting equation (S5) equal to equation (S6). Specifically, equation (S5) is solved for a fixed k_{skin} and $\rho_{skin}c_{p,skin}$. equation (S6) is then solved in an iterative fashion to minimize the error between equation (S6) and equation (S5), where *r(t)* is allowed to vary, and k_{skin} and $\rho_{skin}c_{p,skin}$ are fixed to the values used in the solution for equation (S5). k_{skin} = 0.35 W m^{-1} K^{-1} and $\rho_{skin}c_{p,skin}$ = 2.33 J cm^{-3} K^{-1} are the approximate midpoint values of the *in vivo* data, and are used to establish *r(t)* for the three sensor distances of ~3.5 mm, ~4.7 mm, and ~5.8 mm. *r(t)* begins at a value near that of the distance between the heat source and nearest edge of the sensor, and rapidly approaches the mean sensor distance from the heater. *r(t)* is, more generally, a function of $\rho_{skin}c_{p,skin}t/k_{skin}$, and the solutions of *r(t)* for k_{skin} = 0.35 W m^{-1} K^{-1} and $\rho_{skin}c_{p,skin}$ = 2.33 J cm^{-3} K^{-1} are shown in Figs. S7*A*-*C*. While *r(t)* is a function of thermal properties as well as time, the *r(t)* values shown in Figs. S7**a**-**c** are

assumed to be reasonable approximations for all thermal properties encountered on skin *in vivo*. The error associated with this approximation can be estimated by determining *r(t)* for one set of thermal property values (the mid-range values of the *in vivo* data), and equation (S5) is solved for a set of thermal property values different from those used to determine *r(t)* (high-range values of the *in vivo* data). Equation (S6) is then solved, where *r(t)* is fixed and k_{skin} and $\rho_{skin}c_{p,skin}$ are varied iteratively to minimize the error between equation (S6) and equation (S5). A typical result from this type of analysis is shown in Fig. S7**d**, along with the results determined by replacing *r(t)* with different time independent values (geometric mean, harmonic mean, and r_1). The discrepancy between the results determined by equation (S5) and the approximation using *r(t)* with equation (S6) are found to be <1%. The still simpler solution using a single, time-independent value in place of *r(t)* are found to produce errors <5%, if chosen appropriately.

References

1. Webb RC, Bonifas AP, Behnaz A, Zhang YH, Yu KJ, et al. (2013) Ultrathin conformal devices for precise and continuous thermal characterization of human skin. Nature Materials 12: 938-944.
2. Gustafsson SE (1991) Transient plane source techniques for thermal conductivity and thermal diffusivity measurements of solid materials. Review of Scientific Instruments 62: 797-804.

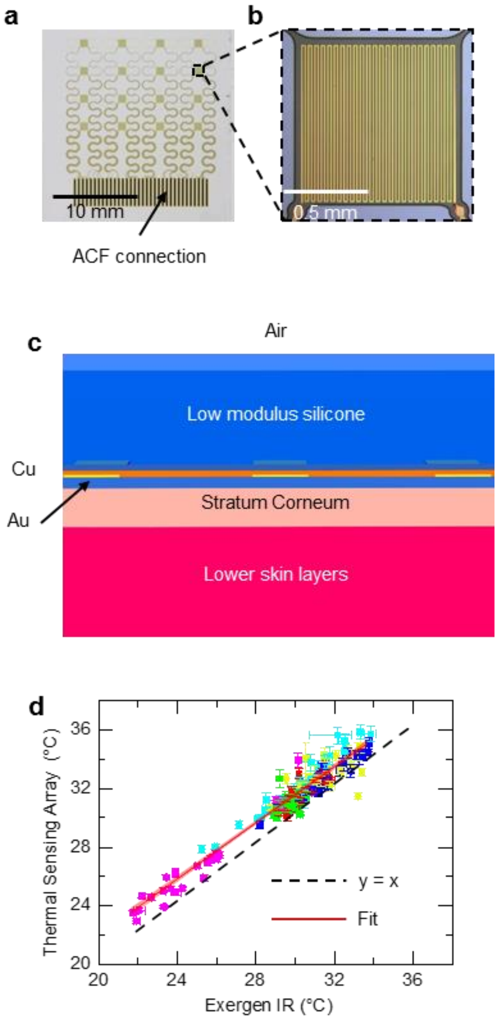

Supplemental Figure S1: **Device construction and temperature comparison to IR measurements.** (**a**) Optical image of 4x4 thermal sensing array, showing the bonding location of the thin, flexible cable (ACF connection). (**b**) Magnified image of a single sensor/actuator element, showing the 10 μm wide, serpentine configuration. (**c**) Cross-sectional schematic showing the device layout on skin. (**d**) Comparison of temperature device readings on six body locations on each of twenty-five subjects, as compared to IR measurements. Pearson correlation coefficient = 0.98.

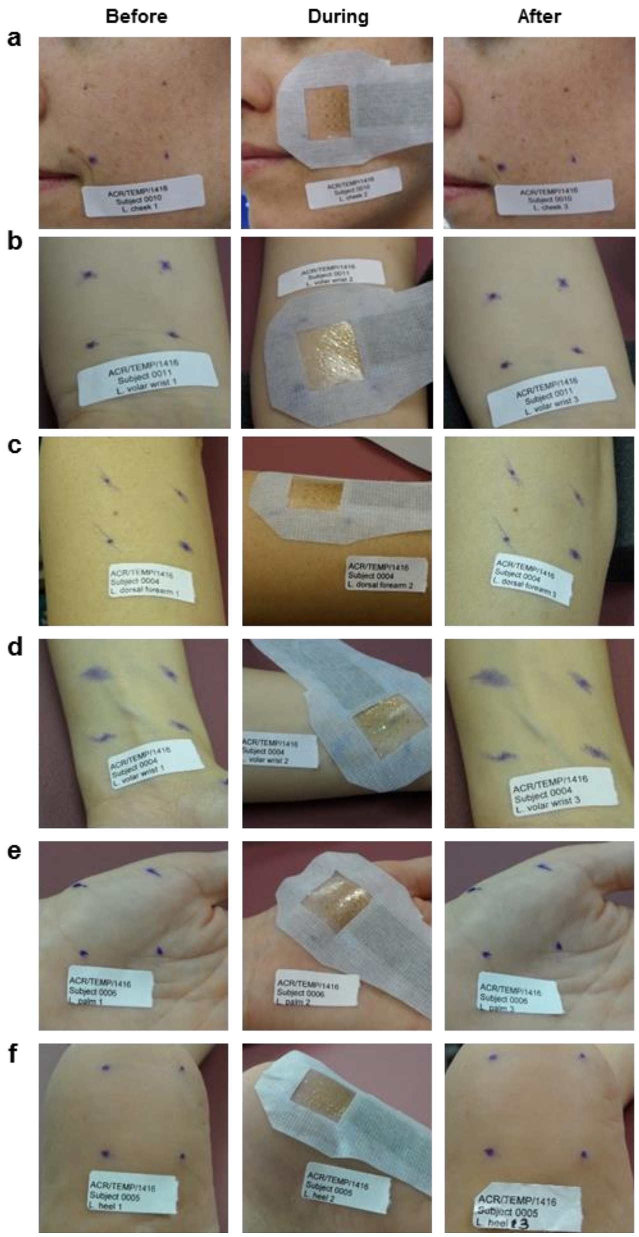

Supplemental Figure S2: **Representative photographs of each body location before, during, and after measurements.** Images show each body location before application of the thermal sensing array, with the device applied to skin during heating applications for thermal measurements, and then after device removal. No irritation is observed as a result of heating, or wearing the device. Body locations are (**a**) cheek, (**b**) volar forearm, (**c**) dorsal forearm, (**d**) wrist, (**e**) palm, and (**f**) heel.

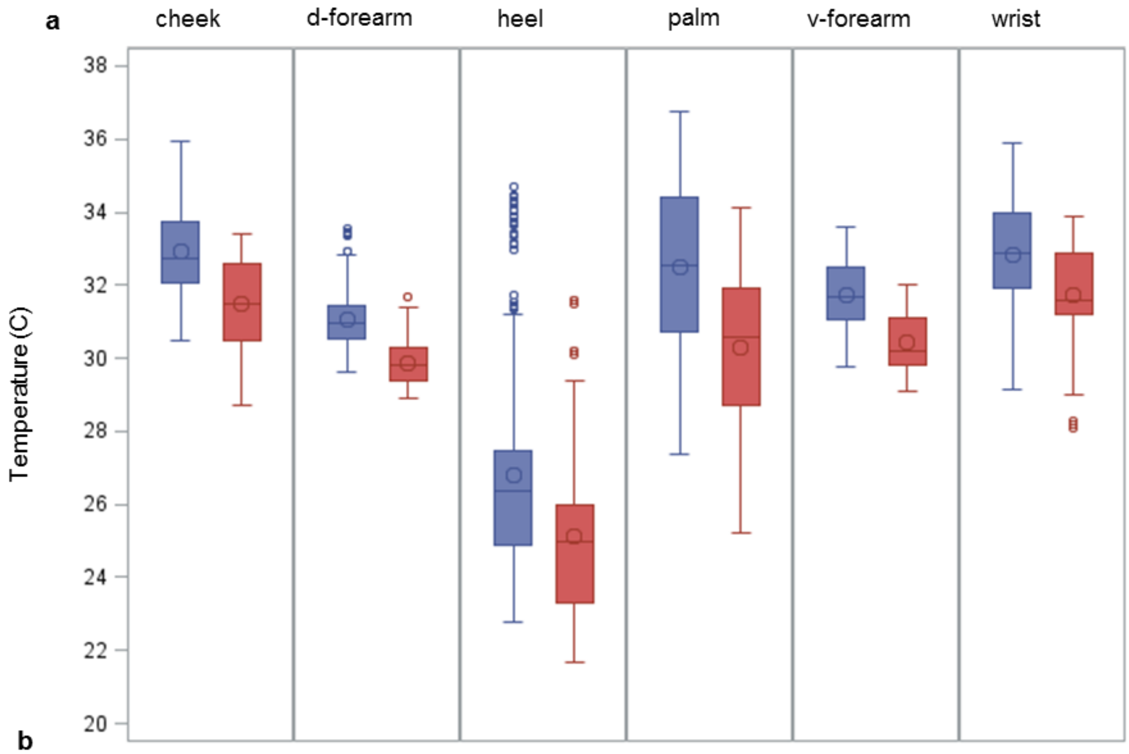

b

Thermal Sensing Array		Intersubject variance	Intrasubject variance
	Cheek	1.31	0.19
	d-Vorearm	0.40	0.12
	Heel	8.34	0.06
	Palm	5.43	0.21
	v-Forearm	0.76	0.07
	Wrist	1.88	0.12
**IR**			
	Cheek	1.48	0.15
	d-Vorearm	0.34	0.02
	Heel	6.90	0.02
	Palm	4.89	0.10
	v-Forearm	0.53	0.03
	Wrist	1.97	0.02

Supplemental Figure S3: Temperature variations across body locations. (**a**) Variation in temperature data between different subjects on different body locations for thermal sensing array (blue) and IR thermometer (red). (**b**) Inter- and intra-subject variance for the thermal sensing array and IR thermometer.

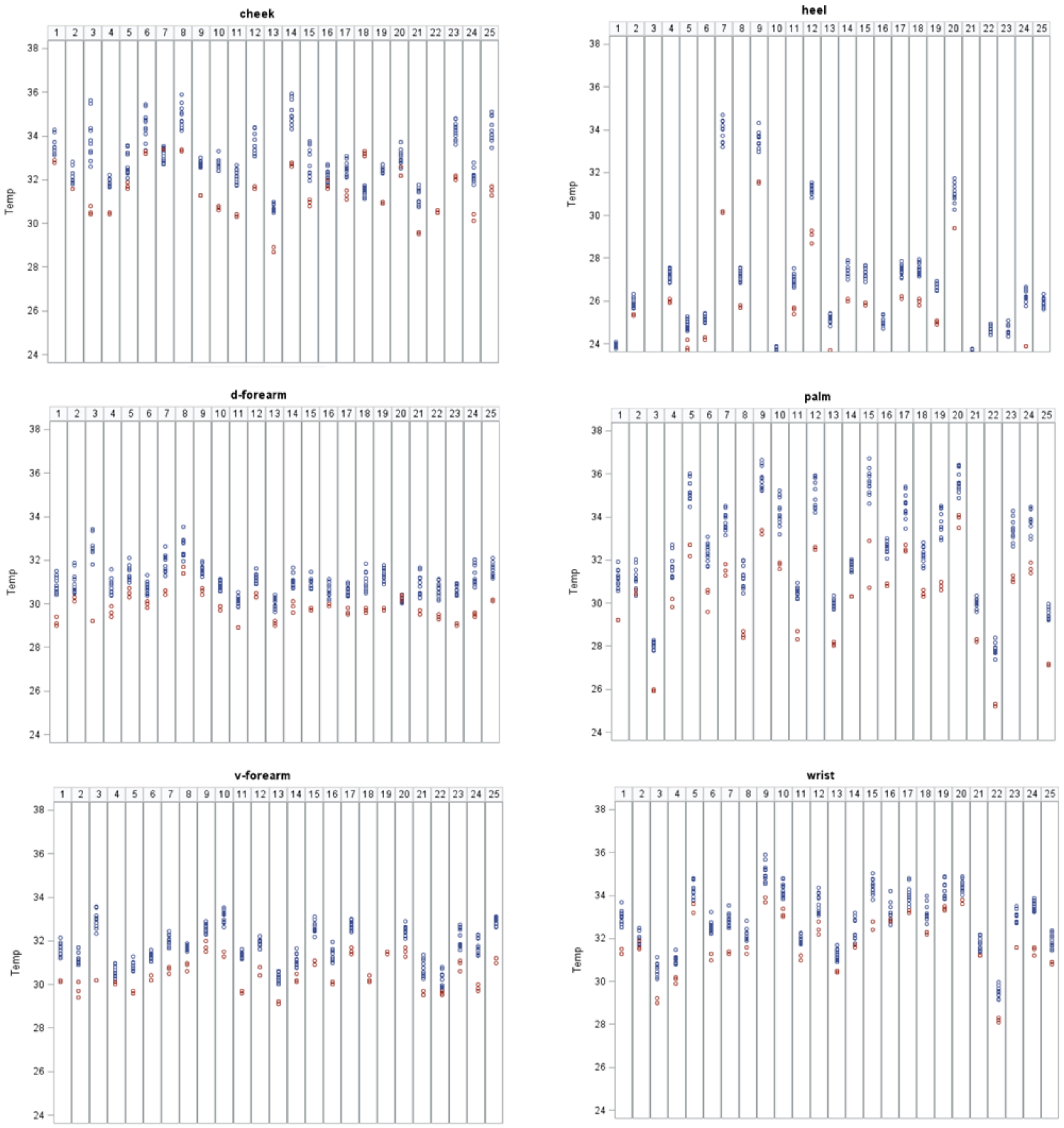

Supplemental Figure S4: Temperature variations across body locations for each subject. Variation in temperature data between different subjects on different body locations for thermal sensing array (blue) and IR thermometer (red).

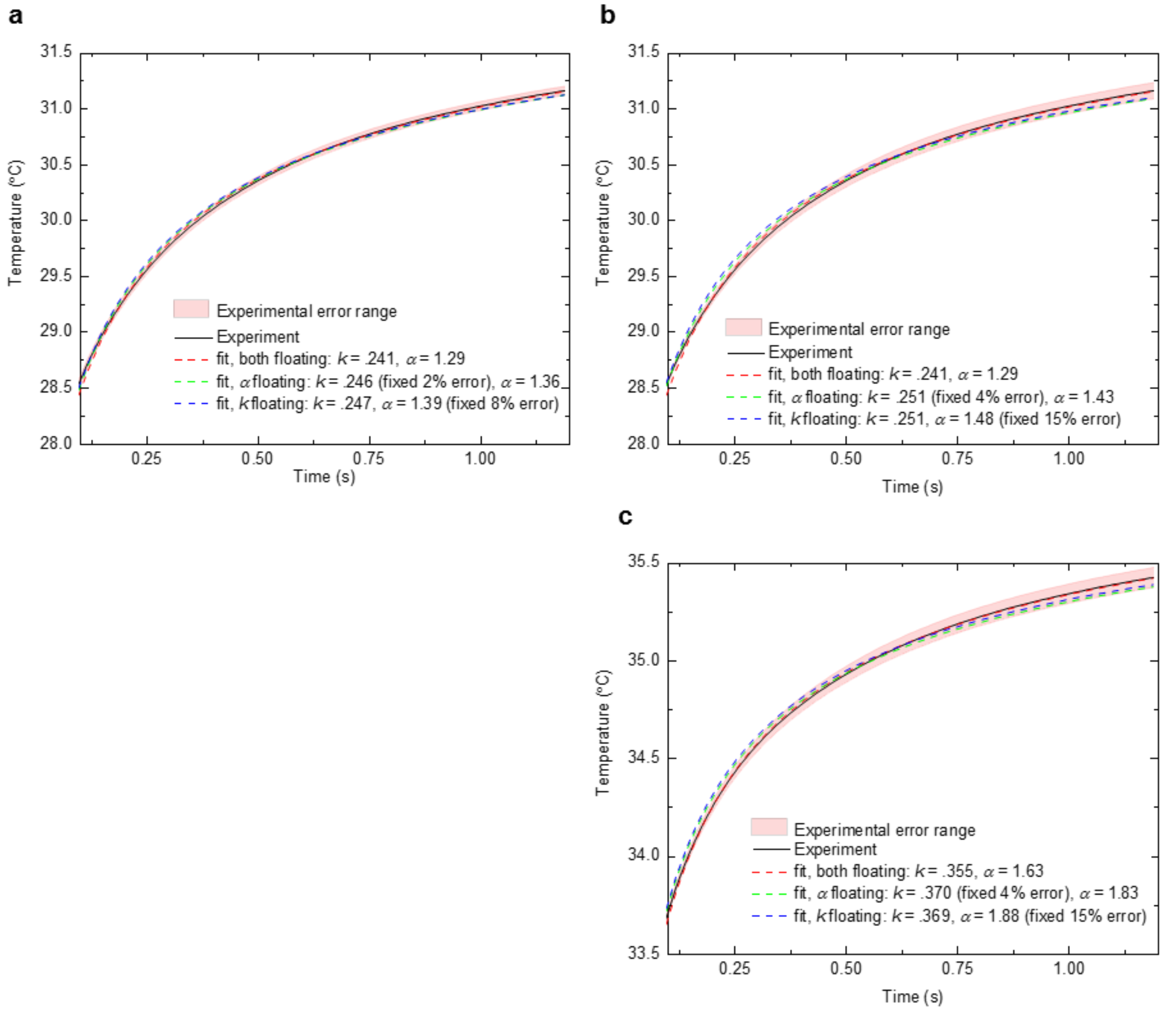

Supplemental Figure S5: Analysis of fitting process sensitivity with experimental error. (**a**) Experimental precision fitting error analysis of representative *in vivo* data on a human heel. Experimental error range is given by 3x the standard deviation of temperature readings from the mean. (**b**) Experimental accuracy fitting error analysis of representative *in vivo* data on a human heel and (**c**) a human cheek. Experimental error range is given by the 95% confidence interval of temperature readings due to calibration errors.

a

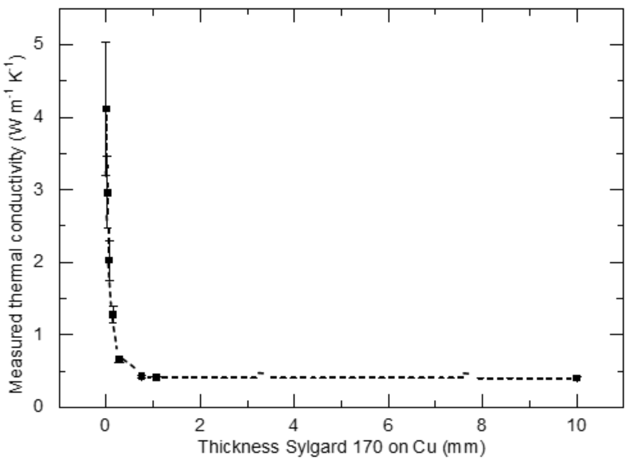

Supplemental Figure S6: **Experimental determination of measurement probing depth.** Measured thermal conductivities by the thermal sensing array for different thickness of a silicone with thermal properties similar to skin (Sylgard 170, Dow Corning, USA; k = 0.39 W m^{-1} K^{-1}, ρ = 1370 kg m^{-3}) on copper. The measured thermal conductivity rises rapidly when the silicone layer becomes thinner than the probing depth, which is given by Eq. **2** to be approximately 0.5 mm.

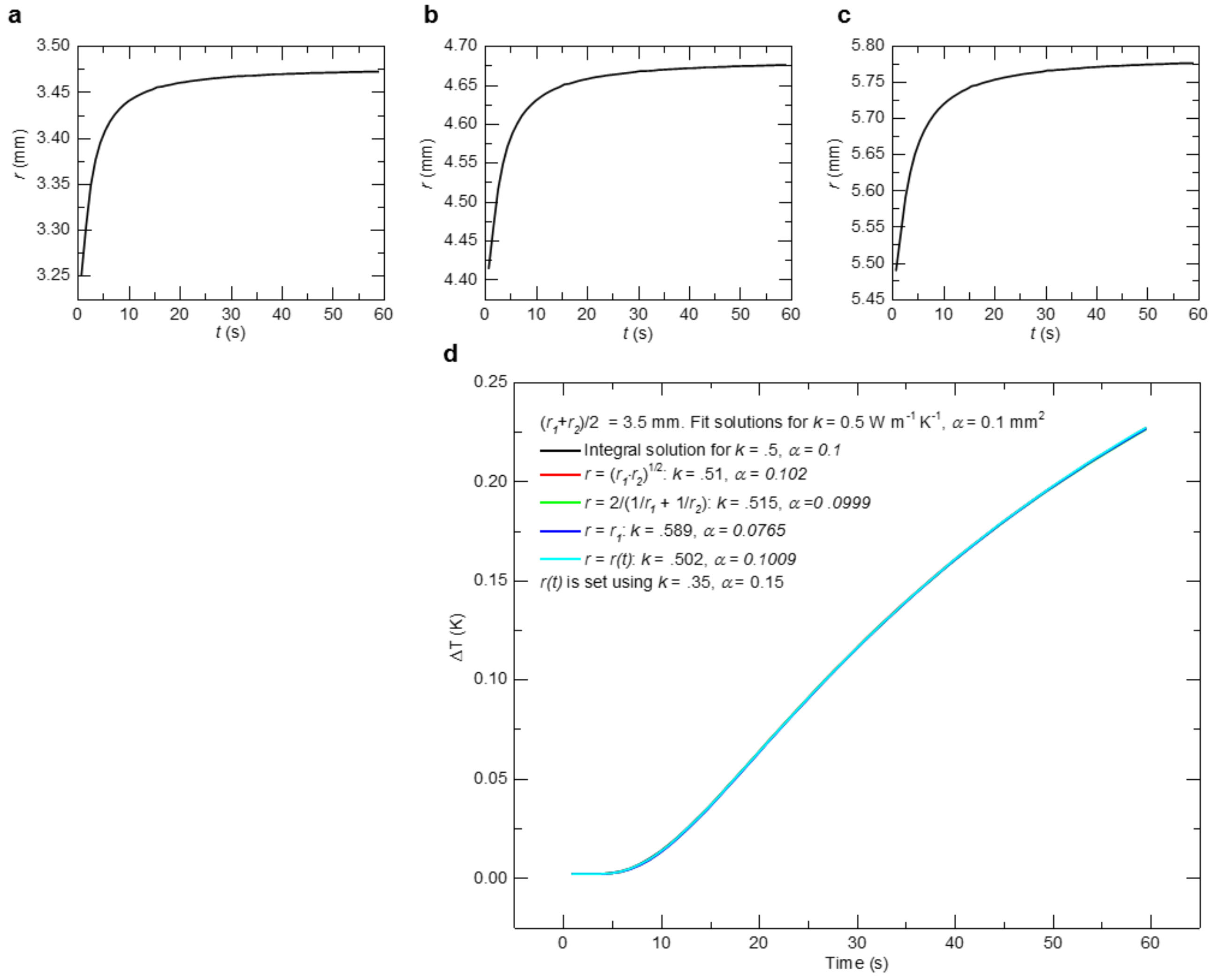

Supplemental Figure S7: **Solutions for *r(t)*.** Numerically determined solutions for *r(t)* over the appropriate measurement time, determined using k = 0.35 W m^{-1} K^{-1} and α = 0.15 mm^2 s^{-1}, for (**a**) r = ~3.5 mm, (**b**) r = ~4.7 mm, and (**c**) r = ~5.8 mm. (**d**) Example temperature rise solutions for a sensor ~3.5 mm away using the integrated solution of Eq. **S5**, *r(t)* given in **a** with Eq. **S6**, and various time independent values of r with Eq. **S6**. *r(t)* gives the smallest discrepancy with Eq. **S5** at <1%, and time independent average values of r give discrepancies <5%.

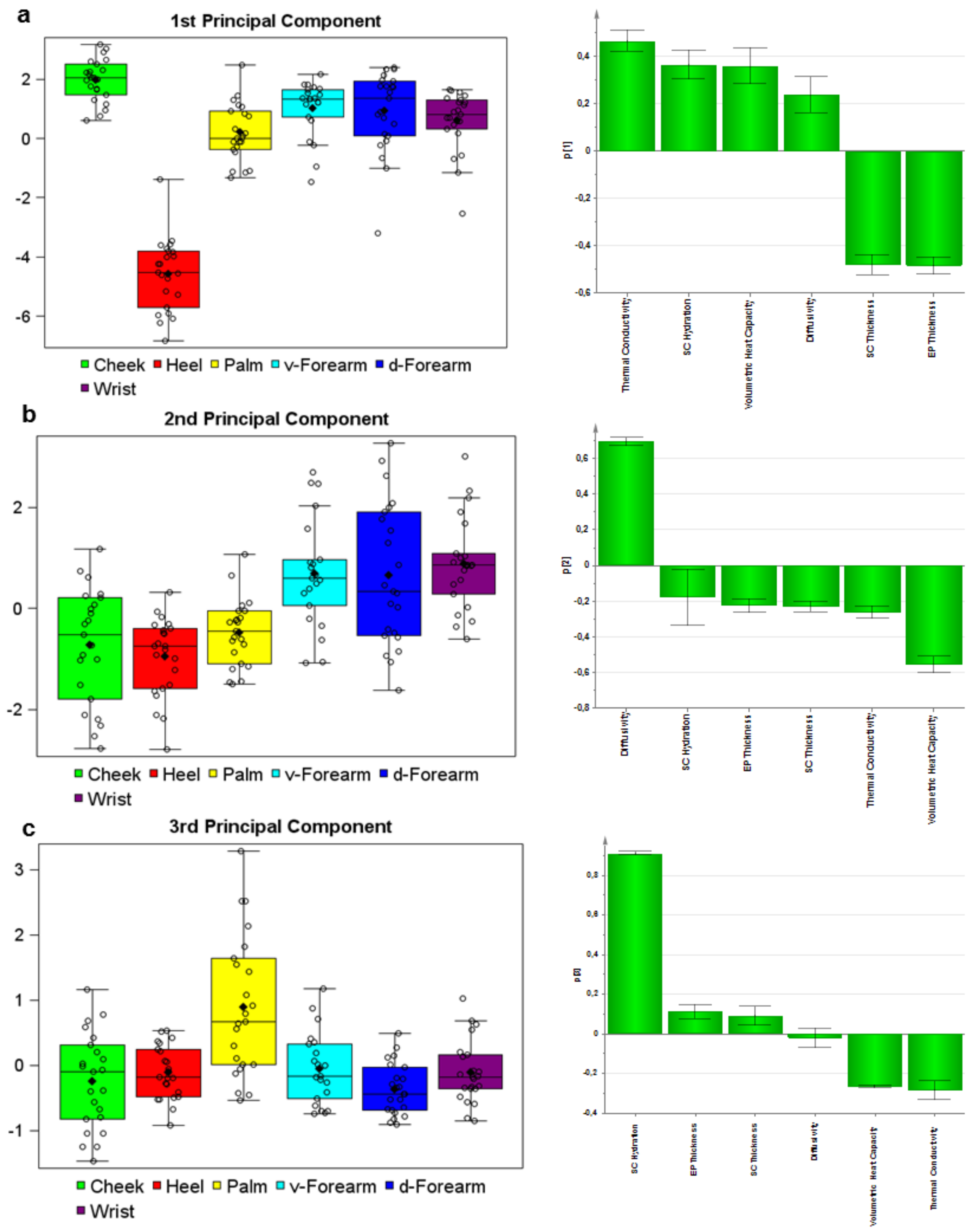

Supplemental Figure S8. Principle component analysis. Boxplot representation of principal components by body location, and their corresponding relation to measured parameters. (**a**) Box plots and correlation weights of the first principal component, (**b**) the second principal component and (**c**) the third principal component.

Multivariate

Correlations

	SC Hydration	SC Thickness	EP Thickness	Thermal Conductivity	Volumetric Heat Capacity	Diffusivity
SC Hydration	1.0000	-0.5523	-0.5479	0.5779	0.5157	0.1376
SC Thickness	-0.5523	1.0000	0.9957	-0.7427	-0.4653	-0.6446
EP Thickness	-0.5479	0.9957	1.0000	-0.7567	-0.4775	-0.6465
Thermal Conductivity	0.5779	-0.7427	-0.7567	1.0000	0.9040	0.1774
Volumetric Heat Capacity	0.5157	-0.4653	-0.4775	0.9040	1.0000	-0.2551
Diffusivity	0.1376	-0.6446	-0.6465	0.1774	-0.2551	1.0000

There are 2 missing values. The correlations are estimated by REML method.

Multivariate Location=cheek

Correlations

	SC Hydration	SC Thickness	EP Thickness	Thermal Conductivity	Volumetric Heat Capacity	Diffusivity
SC Hydration	1.0000	0.0000	0.1456	0.1504	0.2395	-0.2964
SC Thickness	0.0000	0.0000	0.0000	0.0000	0.0000	0.0000
EP Thickness	0.1456	0.0000	1.0000	0.0876	0.1772	-0.2219
Thermal Conductivity	0.1504	0.0000	0.0876	1.0000	0.9418	-0.7469
Volumetric Heat Capacity	0.2395	0.0000	0.1772	0.9418	1.0000	-0.9247
Diffusivity	-0.2964	0.0000	-0.2219	-0.7469	-0.9247	1.0000

Multivariate Location=d-forearm

Correlations

	SC Hydration	SC Thickness	EP Thickness	Thermal Conductivity	Volumetric Heat Capacity	Diffusivity
SC Hydration	1.0000	0.0000	-0.0561	0.7388	0.7431	-0.5789
SC Thickness	0.0000	0.0000	0.0000	0.0000	0.0000	0.0000
EP Thickness	-0.0561	0.0000	1.0000	0.0376	0.0217	0.0334
Thermal Conductivity	0.7388	0.0000	0.0376	1.0000	0.9746	-0.7246
Volumetric Heat Capacity	0.7431	0.0000	0.0217	0.9746	1.0000	-0.8573
Diffusivity	-0.5789	0.0000	0.0334	-0.7246	-0.8573	1.0000

Multivariate Location=heel

Correlations

	SC Hydration	SC Thickness	EP Thickness	Thermal Conductivity	Volumetric Heat Capacity	Diffusivity
SC Hydration	1.0000	-0.6045	-0.6767	0.5433	0.3940	0.0653
SC Thickness	-0.6045	1.0000	0.9579	-0.4823	-0.3962	0.0620
EP Thickness	-0.6767	0.9579	1.0000	-0.5074	-0.4049	0.0434
Thermal Conductivity	0.5433	-0.4823	-0.5074	1.0000	0.9496	-0.5243
Volumetric Heat Capacity	0.3940	-0.3962	-0.4049	0.9496	1.0000	-0.7628
Diffusivity	0.0653	0.0620	0.0434	-0.5243	-0.7628	1.0000

Multivariate Location=palm

Correlations

	SC Hydration	SC Thickness	EP Thickness	Thermal Conductivity	Volumetric Heat Capacity	Diffusivity
SC Hydration	1.0000	-0.5413	-0.4691	0.5784	0.4066	0.1606
SC Thickness	-0.5413	1.0000	0.9145	-0.6861	-0.4179	-0.3327
EP Thickness	-0.4691	0.9145	1.0000	-0.5601	-0.3172	-0.3248
Thermal Conductivity	0.5784	-0.6861	-0.5601	1.0000	0.9013	-0.1981
Volumetric Heat Capacity	0.4066	-0.4179	-0.3172	0.9013	1.0000	-0.6021
Diffusivity	0.1606	-0.3327	-0.3248	-0.1981	-0.6021	1.0000

Multivariate Location=v-forearm

Correlations

	SC Hydration	SC Thickness	EP Thickness	Thermal Conductivity	Volumetric Heat Capacity	Diffusivity
SC Hydration	1.0000	1.0000	-0.0608	0.1426	0.1718	-0.1883
SC Thickness	1.0000	1.0000	-0.0608	0.1426	0.1718	-0.1883
EP Thickness	-0.0608	-0.0608	1.0000	-0.4181	-0.3845	0.2396
Thermal Conductivity	0.1426	0.1426	-0.4181	1.0000	0.9587	-0.6740
Volumetric Heat Capacity	0.1718	0.1718	-0.3845	0.9587	1.0000	-0.8546
Diffusivity	-0.1883	-0.1883	0.2396	-0.6740	-0.8546	1.0000

There are 2 missing values. The correlations are estimated by REML method.

Multivariate Location=wrist

Correlations

	SC Hydration	SC Thickness	EP Thickness	Thermal Conductivity	Volumetric Heat Capacity	Diffusivity
SC Hydration	1.0000	0.0000	-0.2143	0.4363	0.4167	-0.2230
SC Thickness	0.0000	0.0000	0.0000	0.0000	0.0000	0.0000
EP Thickness	-0.2143	0.0000	1.0000	-0.1626	-0.0179	-0.3725
Thermal Conductivity	0.4363	0.0000	-0.1626	1.0000	0.9659	-0.4868
Volumetric Heat Capacity	0.4167	0.0000	-0.0179	0.9659	1.0000	-0.6934
Diffusivity	-0.2230	0.0000	-0.3725	-0.4868	-0.6934	1.0000

Supplemental Table 1. Pearson Correlation coefficients for the correlation analyses (Figs. 4-6).
