# Supplementary figures and images for: Thermal Transport Characteristics of Human Skin Measured In Vivo Using Ultrathin Conformal Arrays of Thermal Sensors and Actuators
Source: PLoS One. 2015 Feb 6;10(2):e0118131. doi: 10.1371/journal.pone.0118131 (PMC4319855; doi:10.1371/journal.pone.0118131)

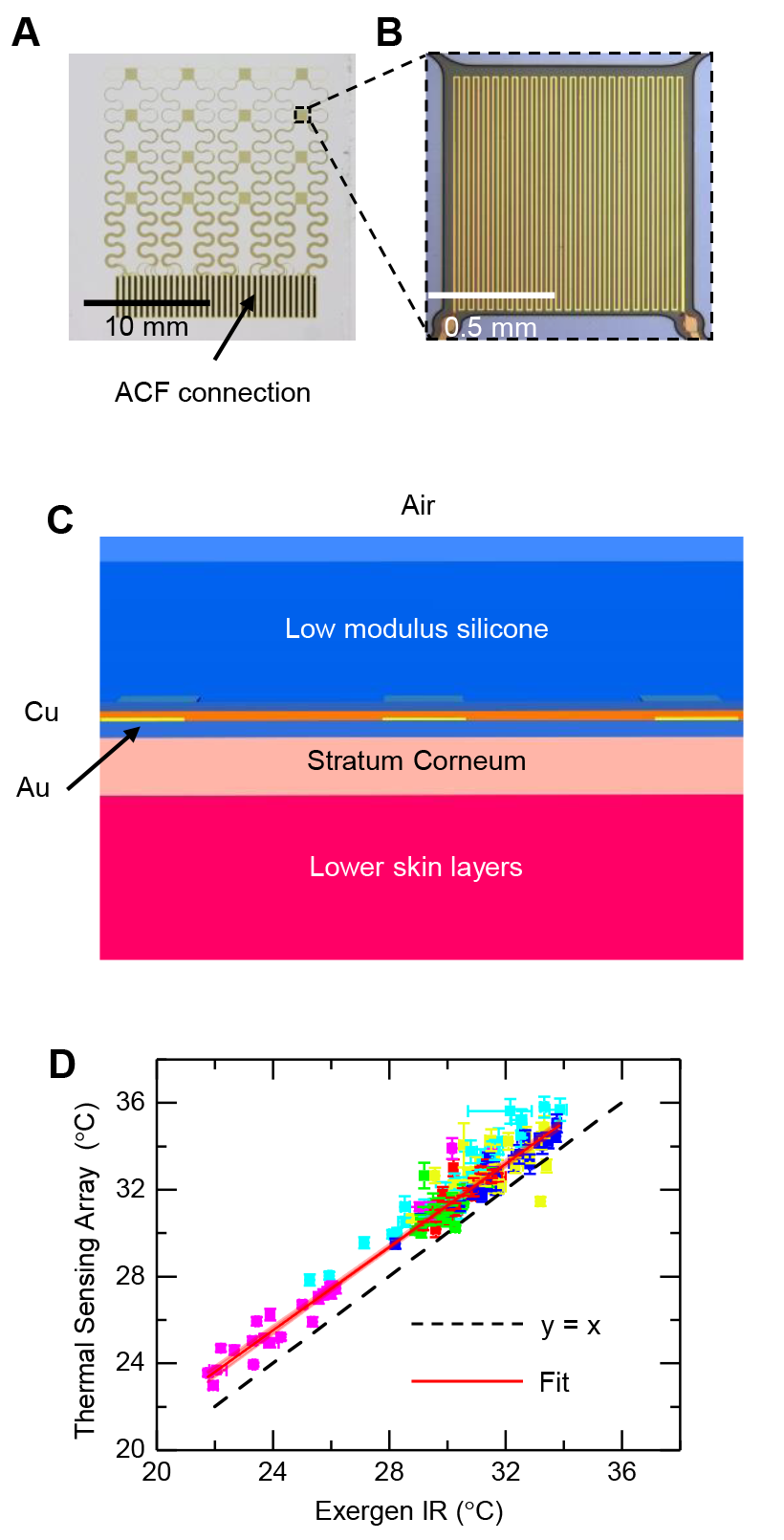

Supplement: S1 Fig — (a) Optical image of 4x4 thermal sensing array, showing the bonding location of the thin, flexible cable (ACF connection). (b) Magnified image of a single sensor/actuator element, showing the 10 μm wide, serpentine configuration. (c) Cross-sectional schematic showing the device layout on skin. (d) Comparison of temperature device readings on six body locations on each of twenty-five subjects, as compared to IR measurements. Pearson correlation coefficient = 0.98. (TIF) [file pone.0118131.s002.tif]

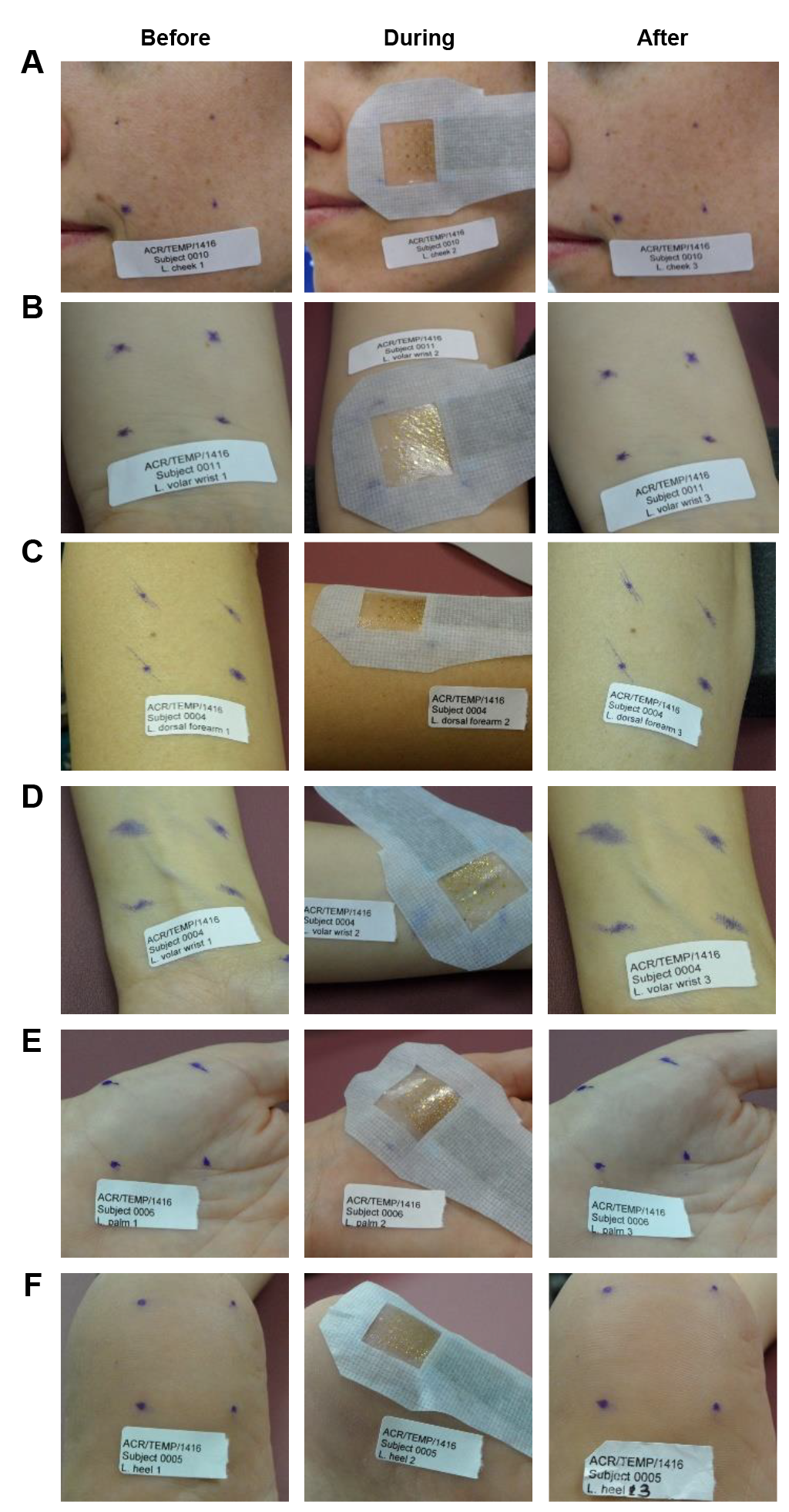

Supplement: S2 Fig — Images show each body location before application of the thermal sensing array, with the device applied to skin during heating applications for thermal measurements, and then after device removal. No irritation is observed as a result of heating, or wearing the device. Body locations are (a) cheek, (b) volar forearm, (c) dorsal forearm, (d) wrist, (e) palm, and (f) heel. (TIF) [file pone.0118131.s003.tif]

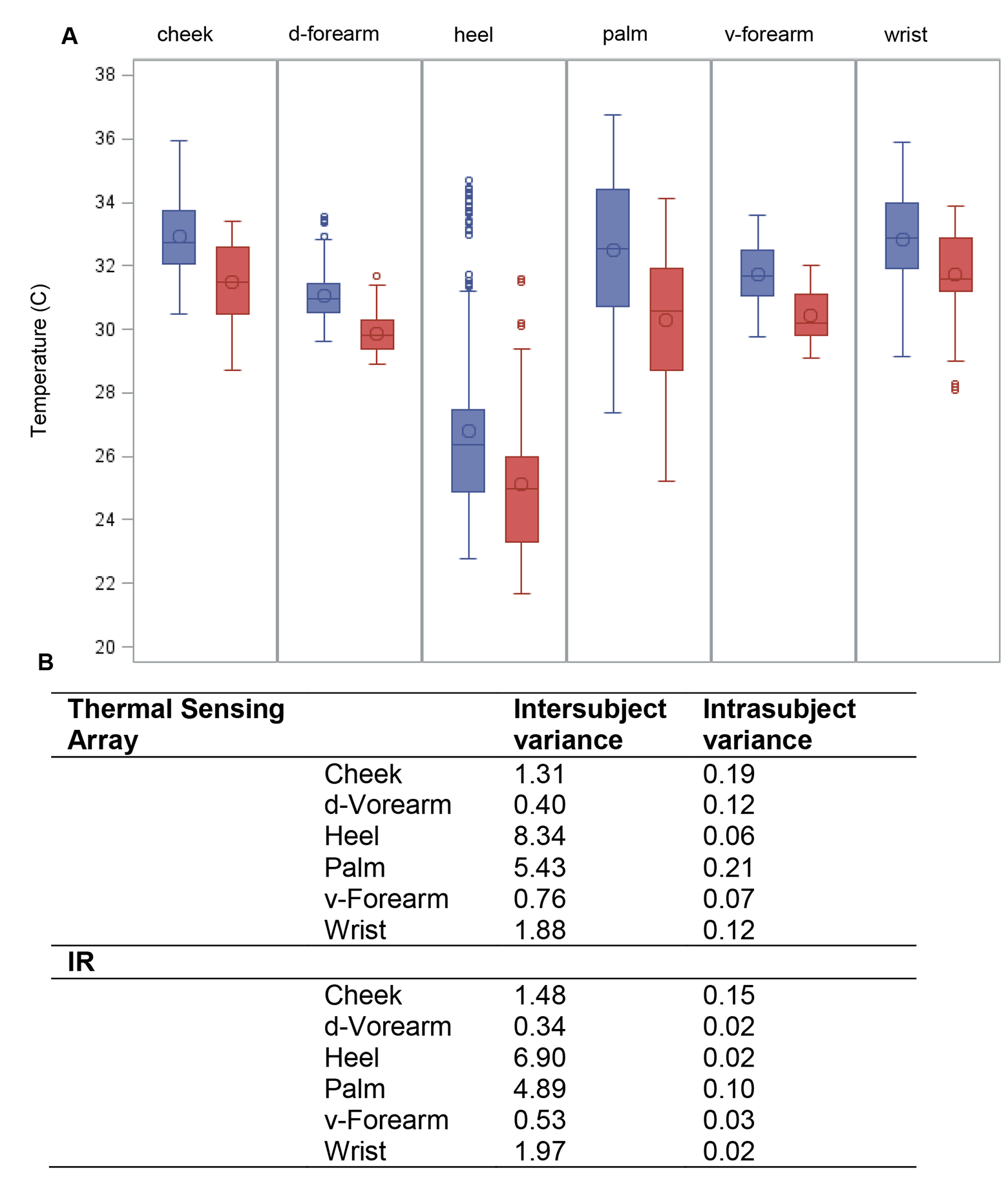

Supplement: S3 Fig — (a) Variation in temperature data between different subjects on different body locations for thermal sensing array (blue) and IR thermometer (red). (b) Inter- and intra-subject variance for the thermal sensing array and IR thermometer. (TIF) [file pone.0118131.s004.tif]

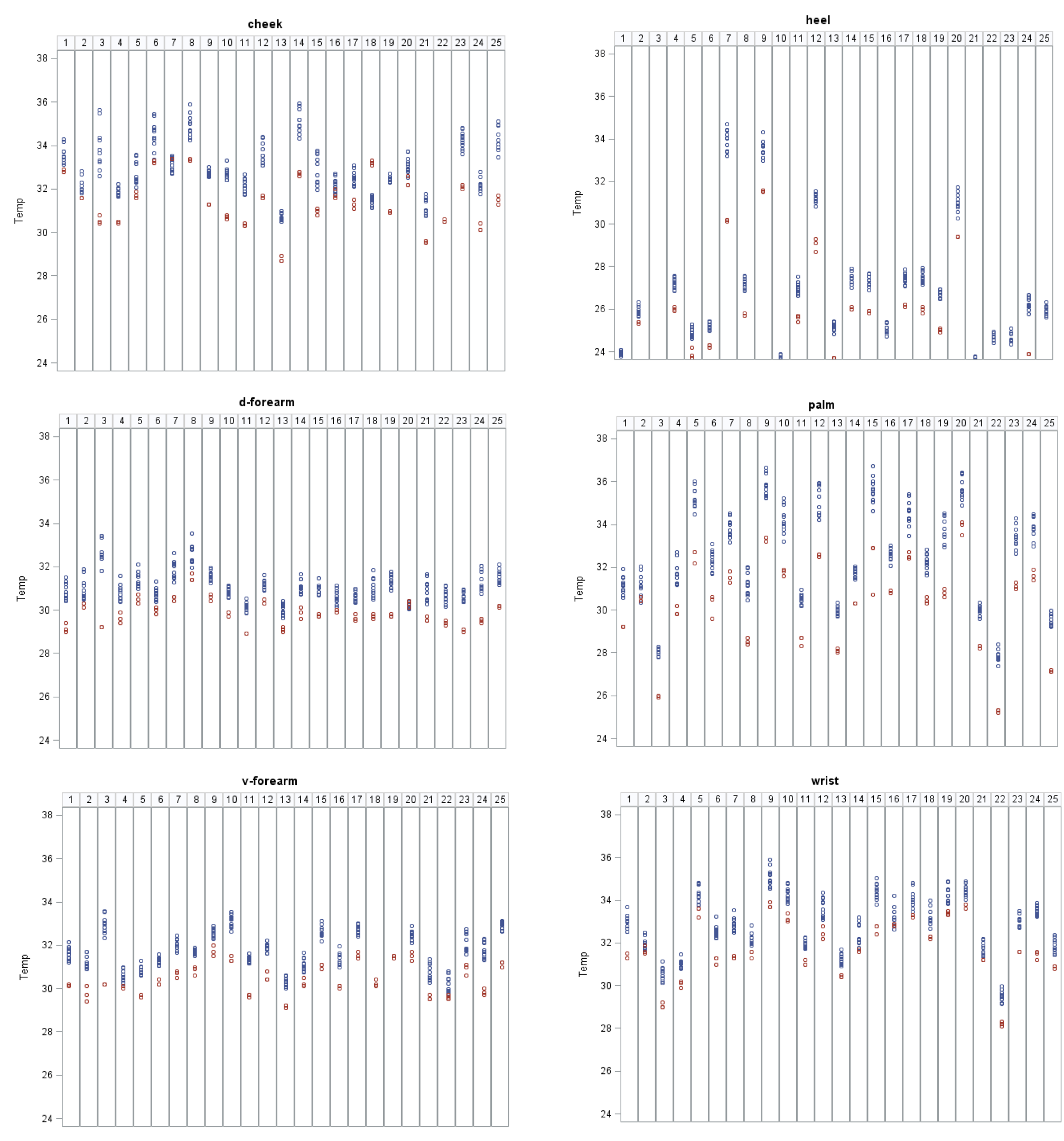

Supplement: S4 Fig — Variation in temperature data between different subjects on different body locations for thermal sensing array (blue) and IR thermometer (red). (TIF) [file pone.0118131.s005.tif]

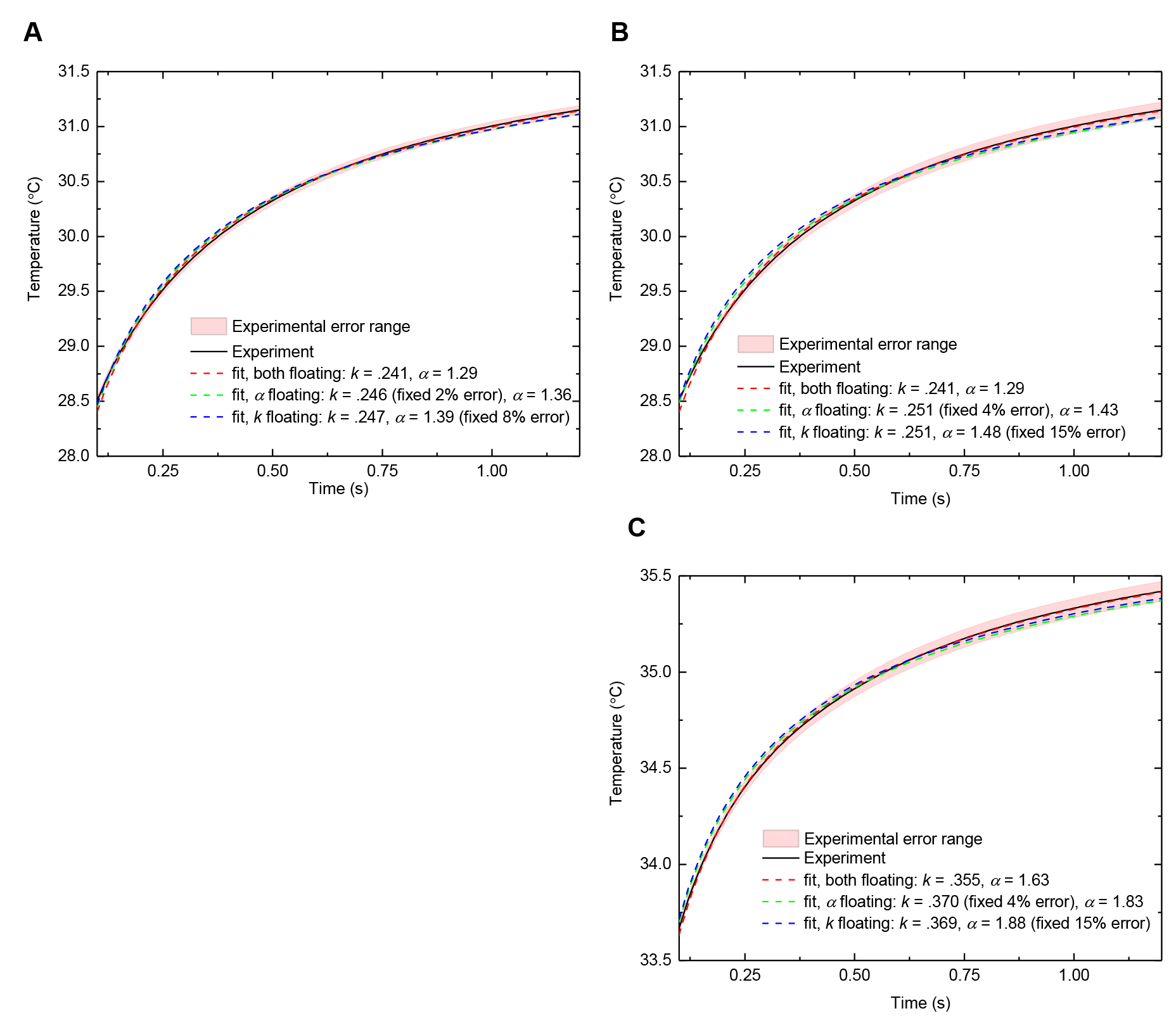

Supplement: S5 Fig — (a) Experimental precision fitting error analysis of representative in vivo data on a human heel. Experimental error range is given by 3x the standard deviation of temperature readings from the mean. (b) Experimental accuracy fitting error analysis of representative in vivo data on a human heel and (c) a human cheek. Experimental error range is given by the 95% confidence interval of temperature readings due to calibration errors. (TIF) [file pone.0118131.s006.tif]

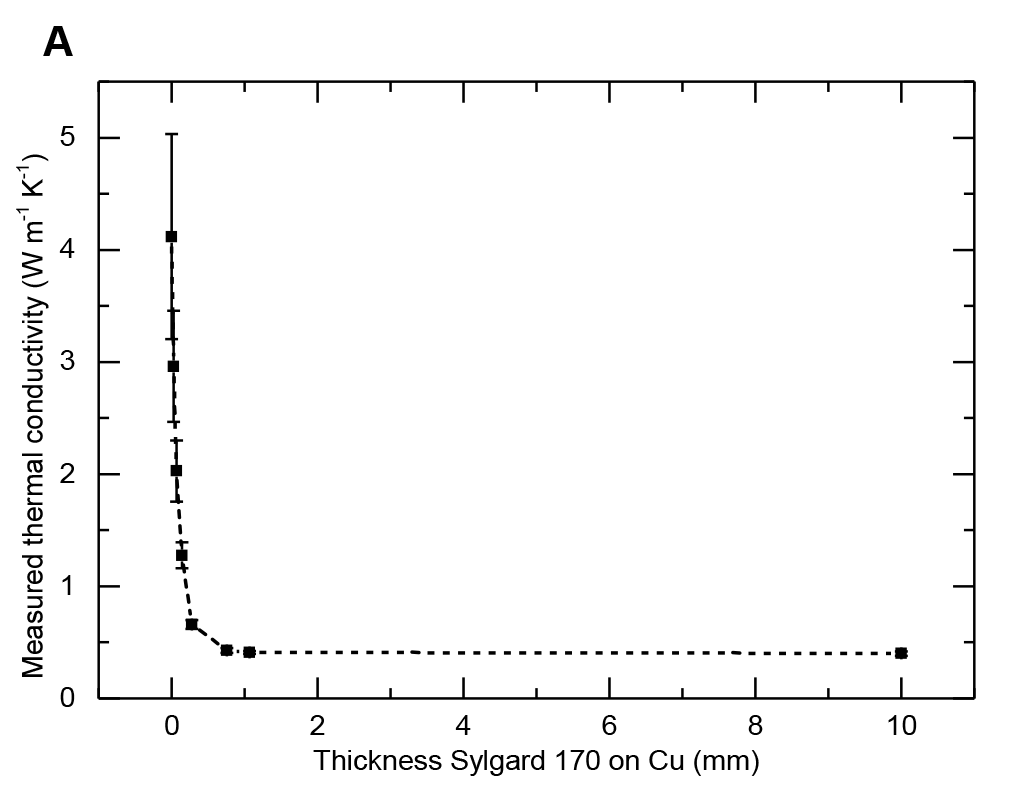

Supplement: S6 Fig — Measured thermal conductivities by the thermal sensing array for different thickness of a silicone with thermal properties similar to skin (Sylgard 170, Dow Corning, USA; k = 0.39 W m-1 K-1, ρ = 1370 kg m-3) on copper. The measured thermal conductivity rises rapidly when the silicone layer becomes thinner than the probing depth, which is given by Eq. 2 to be approximately 0.5 mm. (TIF) [file pone.0118131.s007.tif]

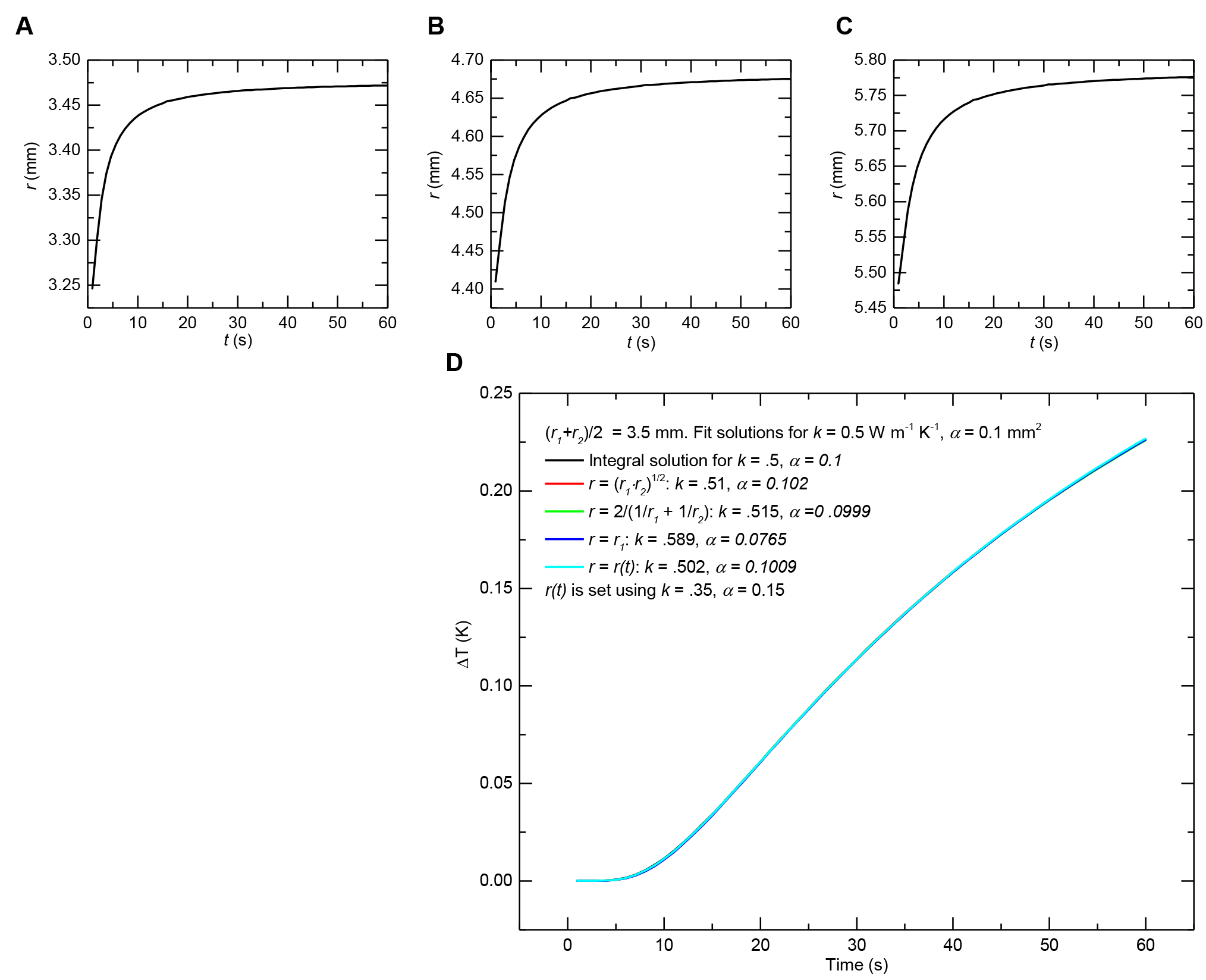

Supplement: S7 Fig — Numerically determined solutions for r(t) over the appropriate measurement time, determined using k = 0.35 W m-1 K-1 and α = 0.15 mm2 s-1, for (a) r = ~3.5 mm, (b) r = ~4.7 mm, and (c) r = ~5.8 mm. (d) Example temperature rise solutions for a sensor ~3.5 mm away using the integrated solution of Eq. S5, r(t) given in a with Eq. S6, and various time independent values of r with Eq. S6. r(t) gives the smallest discrepancy with Eq. S5 at <1%, and time independent average values of r give discrepancies <5%. (TIF) [file pone.0118131.s008.tif]

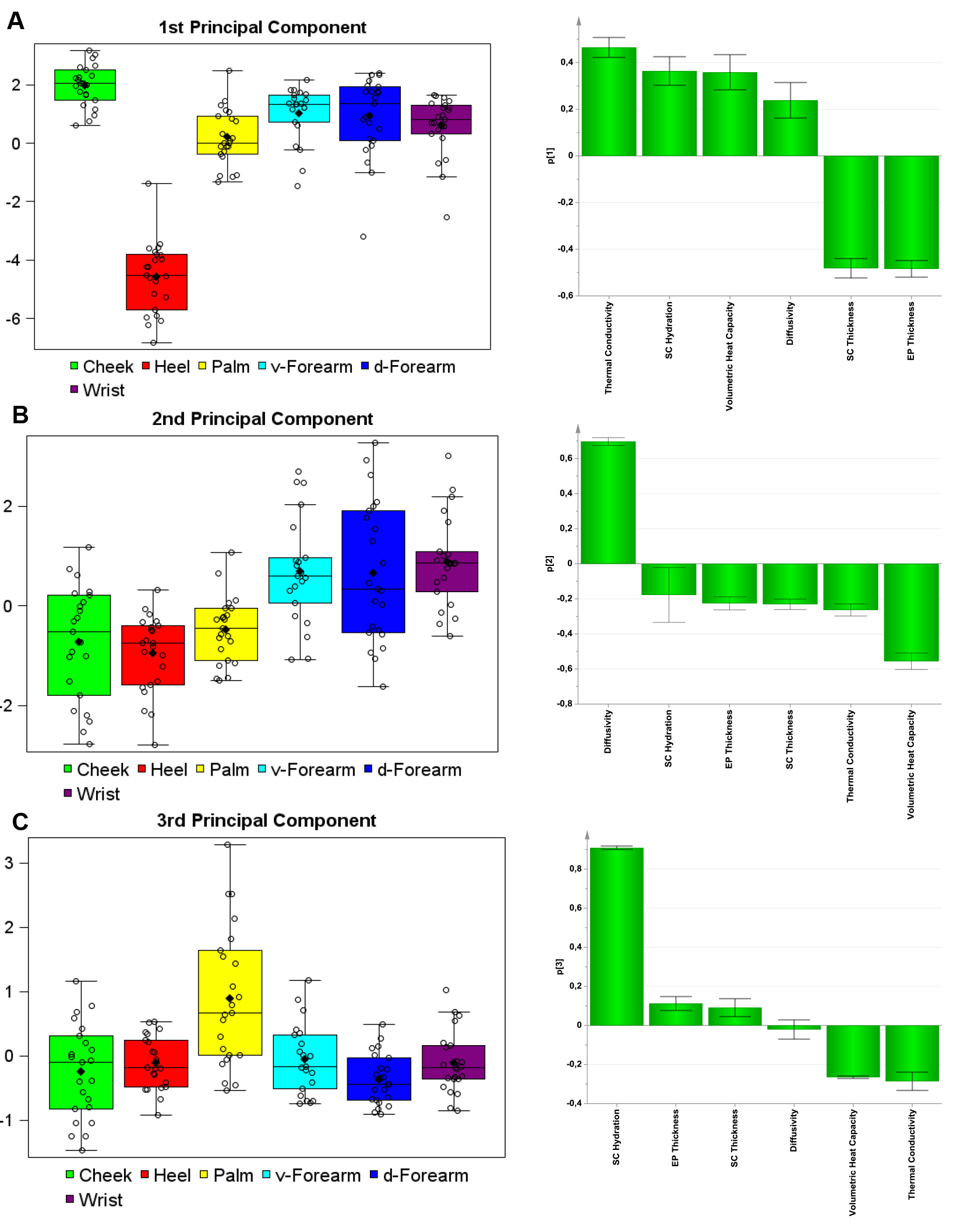

Supplement: S8 Fig — Boxplot representation of principal components by body location, and their corresponding relation to measured parameters. (a) Box plots and correlation weights of the first principal component, (b) the second principal component and (c) the third principal component. (TIF) [file pone.0118131.s009.tif]

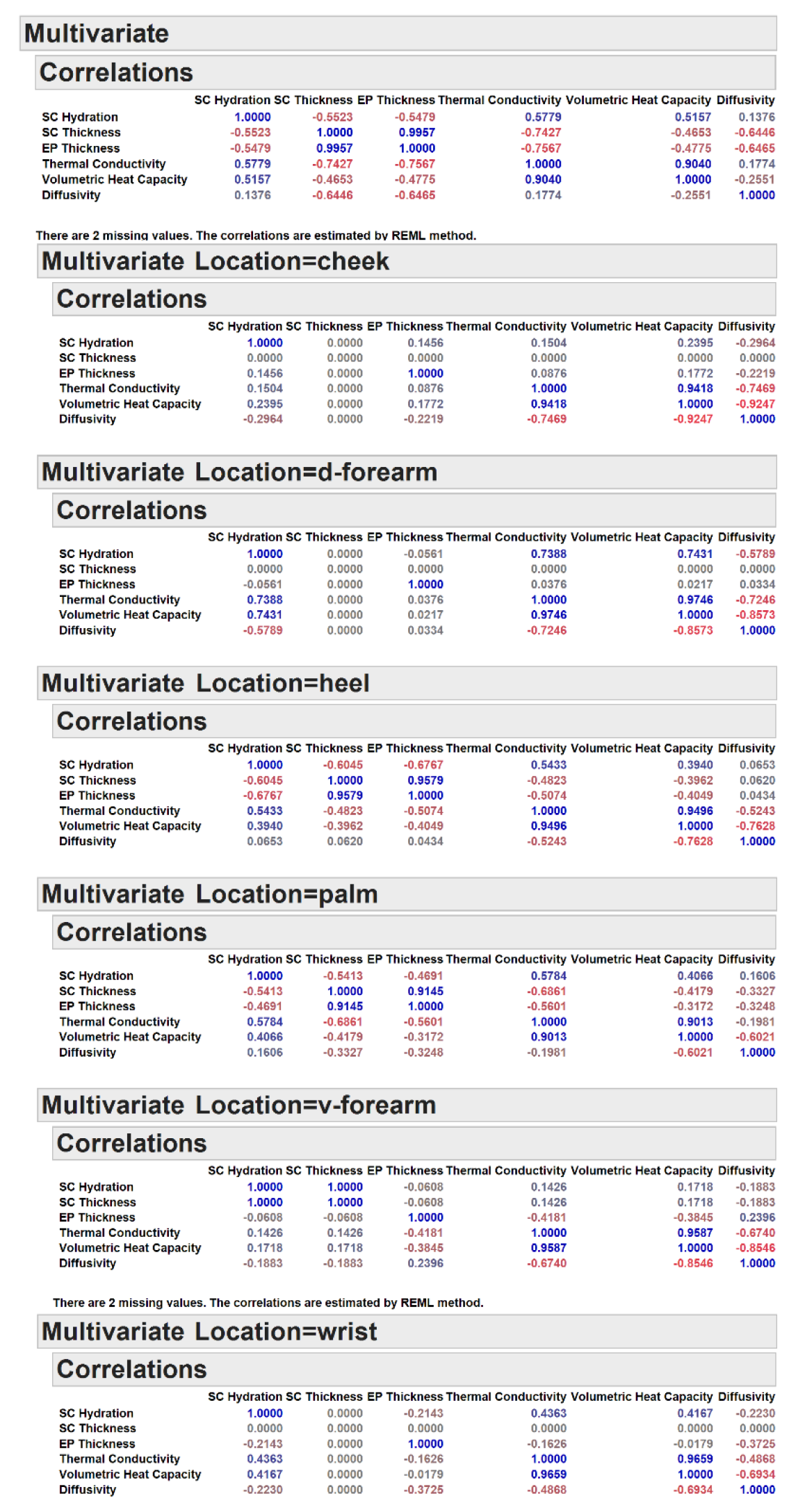

Supplement: S1 Table — (TIF) [file pone.0118131.s010.tif]
